# A remarkable new blue ***Ranitomeya*** species (Anura: Dendrobatidae) with copper metallic legs from open forests of Juruá River Basin, Amazonia

**DOI:** 10.1371/journal.pone.0321748

**Published:** 2025-05-14

**Authors:** Esteban Diego Koch, Alexander Tamanini Mônico, Jussara Santos Dayrell, Anthony Santana Ferreira, Silionamã Pereira Dantas, Jiří Moravec, Albertina Pimentel Lima

**Affiliations:** 1 Programa de Pós-Graduação em Genética, Conservação e Biologia Evolutiva, Instituto Nacional de Pesquisas da Amazônia, Manaus, Amazonas, Brazil; 2 Coordenação de Biodiversidade, Instituto Nacional de Pesquisas da Amazônia, Manaus, Amazonas, Brazil; 3 Department of Zoology, National Museum of the Czech Republic, Czech Republic; CONICET: Consejo Nacional de Investigaciones Cientificas y Tecnicas, ARGENTINA

## Abstract

Poison dart frogs (*Dendrobatidae*) are known for their aposematic coloration and toxic skin, making them a frequent subject of interest and research. However, descriptions of new species of *Ranitomeya* were interrupted for more than a decade. The implementation of a RAPELD (Rapid Assessment surveys of Long-Term Ecological Research) module in the Juruá River basin, a highly biodiverse and underexplored region, led to the record of a *Ranitomeya* species with blue dorsal stripes and coppery limbs. Herein we use morphological, morphometric, advertisement call, natural history, tadpole data and genetic data to describe the new species. Our phylogenetic analysis places the species within the *Ranitomeya vanzolinii* clade, and all delimitation methods confirmed its status as a new species. The species is characterized by its (i) small size (snout-vent length: males 15.2–17.0 mm, females 14.4–16.9 mm), (ii) dorsum with light sky-blue stripes on a reddish-brown ground, and metallic copper limbs with reddish-brown spots, (iii) ring-shaped granular region on the belly, (iv) toes with poorly developed lateral fringes, (v) later tadpole stages with tooth rows P1 = P2 > P3, P3 of 83–87% of P1, and conspicuous light sky-blue dorsal stripes, and (vi) cricket-like advertisement call consisting of 16–35 notes, call duration of 490–1,005 ms, note duration of 8.2–16.9 ms and dominant frequency of 5,168–6,029 Hz. The discovery of the new species emphasizes the significance of researching under-sampled regions like the Juruá River basin, and the usefulness of using a multidisciplinary approach to reveal new dendrobatid species.

## Introduction

Poison Frogs (Dendrobatidae) have always attracted human attention with their bright aposematic color patterns, toxic skin alkaloids and complex parental care behavior [1,2]. Therefore, their biology and taxonomy have been the subject of extensive studies that led and still lead to the description of new species [e.g., 3–9]. Nevertheless, the knowledge of the species diversity within some genera of dendrobatids is not yet complete due to the morphological similarity and high variability of the color patterns of individual species. This is especially the case of the genus *Ranitomeya* Bauer, 1986 that has only 16 currently recognized species and had not seen description of any new species for over a decade [10].

*Ranitomeya* species undergone a diversification bursting around 4–6 MA [11]. They are distributed in the northern part of South America, through the Andean foothills and in the Amazonian rainforest and most of the currently recognized species have a narrow geographic range [4,10]. Some of them appear to have low intraspecific genetic variability and also retain a conserved morphology (low interspecific genetic distances were expected by Pérez-Peña et al. [12]), others have evolved multiple external color patterns [4].

The long-recognized high intraspecific color pattern variation including mimetic systems within *Ranitomeya* was the main reason for taxonomic complexity within this genus [4,13–15]. Traditional taxonomic studies frequently took into account only the characters that agreed with the definition of the individual taxa, focused mainly on the color pattern and omitted more detailed morphological and ecological traits [4]. Such an approach resulted in number of confusions and in erroneous delimitations and descriptions of some new nominal taxa (e.g., *R. ignea* [16]; *R. duellmani* [17]; *R. intermedia* [17]).

Despite the recent clarification of inter and intraspecific relationships within *Ranitomeya* by Muell et al. [11] the taxonomic status of some still unnamed Amazonian dendrobatid populations belonging to the genus *Ranitomeya* remains to be solved. In this regard, methods of integrative taxonomy, combining analyzes of morphological, genetic, bioacoustic, behavioral and ecological data, promise significant progress.

One of such still unnamed *Ranitomeya* population has been recently discovered and monitored in the basin of the Juruá River (a southwestern tributary of the Amazonas River) in the western Brazilian Amazonia. The Juruá River basin is one of the most difficult accessible and least sampled regions in the entire Amazonia [18,19]. It is believed to harbor an unusually high vertebrate diversity, but its remoteness and the hard logistics make difficult the development of long-term studies that allow understanding diversity patterns of this area. Therefore, the implementation of the RAPELD (Rapid Assessment Program for Environmental and Long-Term Ecological Research) research modules [20] in the region represent a great improvement in this field [21]. For amphibians, most records come from specific inventories, especially from the upper waters of Juruá River [e.g., 22–24]. Our preliminary results show that this area has a very high potential for the presence of new taxa, with several unnamed species already identified [e.g., 19,21,25,26].

During our RAPELD sampling expeditions in the Juruá River in 2023 and 2024 we recorded a new species of *Ranitomeya* with sky-blue dorsal stripes and metalized reddish-brown limbs. Herein we describe it as the new species based on morphological, genetic and bioacoustic data and provide information about its advertisement call, courtship call, reproductive behavior, and tadpole morphology.

## Materials and methods

### Sampling

Twenty-six adult individuals of the new species of *Ranitomeya* were collected in the RAPELD sampling module of Comunidade de Nova Esperança (6°42’12.7“S 70°22’12.1”W), Eirunepé municipality, state Amazonas, Brazil. The frogs were anaesthetized and euthanized with topic 5% lidocaine. Their muscle or liver tissue samples were preserved in 100% ethanol for posterior genetic analysis. The voucher specimens were fixed in 10% formalin and preserved in 70% ethanol. Specimens were sexed by observing secondary sexual characters (vocal slits), and gonads through dissection. Vouchers were deposited in the herpetological collection of the Instituto Nacional de Pesquisas da Amazônia – **INPA-H** (Manaus, Brazil) and the Museu Paraense Emílio Goeldi – **MPEG** (Belém, Brazil).

Five tadpoles were collected at same site as adult individuals, euthanized with 5% lidocaine, and fixed and preserved in 5% neutral-buffered formalin, the tip of the tail of three of those specimens were cut and preserved in 100% ethanol for posterior genetic analysis.

Advertisement calls of seven males (INPA-H 47573 – SVL: 15.6 mm, INPA-H 47575 – SVL: 15.9 mm, INPA-H 47576 – SVL: 15.8 mm, INPA-H 47578 – SVL: 15.4 mm, INPA-H 47581 – SVL: 15.7 mm, INPA-H 47586 – SVL mm: 16.2 and INPA-H 47587 – SVL: 15.2 mm) and a courtship call of a single male (MPEG 45225 – SVL: 15.9 mm) were recorded in the vicinity of Nova Esperança, municipality of Eirunepé, state Amazonas, Brazil. Air temperature (25.6–27.5°C) and humidity (89–98%) during call recording were measured with a thermohygrometer Incoterm 7663.02.0.00. Calls were recorded with a digital recorder (PCM-D50, Sony) and a unidirectional microphone (K6/ME66, Sennheiser, Germany). We set recorders at a sampling frequency of 16 kHz and a resolution of 16-bits, storing recordings as WAV files. We recorded approximately 1 min of consecutive advertisement calls with the microphone positioned approximately 1 m from the calling male. Recordings were deposited in the acoustic repository Fonoteca Neotropical Jacques Vielliard – **FNJV** at the University of Campinas (Campinas, Brazil) under accession numbers FNJV 0124340–0124347.

### Morphology

Forty-seven morphometric measurements of 19 adult males and seven adult females of the new species were taken to the nearest 0.01 mm using a Leica stereomicroscope (model S8APO) coupled to a Leica DFC295 camera, except for the SVL that was measured to the nearest 0.1 mm with a digital caliper.

Forty-seven morphometric measurements were taken from eight adult males and five adult females of the new species, following Brown et al. [4] [snout to vent length (**SVL**), head width (**HW**), head length (**HL**), interorbital distance (**IOD**), upper eyelid width (**UEW**), tympanum diameter (**TD**), eye-tympanum distance (**DET**), eye diameter (**ED**), body width (**BW**), knee-knee distance (**KK**), femur length (**FL**), tibia length (**TL**), foot length/ Toe IV length (**FoL**), hand length/ Finger III length (**HaL**), fingers I (**L1F**) and II (**L2F**) length, Finger III disc width (**W3FD**), finger width just below III (**W3F**)], Watters et al. [27] [snout length (**SL**), eye-nostril distance (**END**), internarial distance (**IND**), tarsus length (**TaL**), arm length (**AL**), forearm length (**FAL**), Finger IV length (**L4F**); toes I (**L1T**), III (**L3T**) and V (**L5T**) length, Toe IV disc width (**W4TD**), fingers II (**W2FD**) and IV (**W4FD**) discs width, Finger IV width just below disc (**W4F**)], and Serrano et al. [28] [snout-nostril distance (**TSCN**), mouth-tympanic distance (**MTD**), Toe III disc width (**W3TD**), toes III (**W3T**) and IV (**W4T**) width just below disc]. Besides these, we also include Toe II length (**L2T**), toes I (**W1TD**), II (**W2TD**) and V (**W5TD**) disc width, toe I (**W1T**), II (**W2T**) and V (**W5T**) width just below disc, Finger I disc width (**W1FD**), and fingers I (**W1F**) and II (**W2F**) width just below disc.

The format of the description and terminology of the morphological characters follow Kok and Kalamandeen [29] and Brown et al. [4]. Color in life was described based on photographs taken in the field, following the color catalog provided by Köhler [30]. For a schematic draw explaining the methods of measurements see S1 Fig. Morphological raw data obtained for the adult specimens are provided in S1 Table.

### Bioacoustics

Bioacoustic variables were analyzed with Raven Pro 1.6 [31] with the following configuration: window = Blackman, Discrete Fourier Transform = 2,048 samples and 3dB filter bandwidth = 80.0 Hz. The following temporal and spectral traits were measured: call duration (**CD**), number of notes per call (**NN**), silence between calls - (**SBC**), note duration (**ND**), silence between notes (**SBN**), and minimum frequency (**LF**), maximum frequency (**HF**), and dominant frequency (**DF**). Dominant frequency was measured using the *Peak frequency* function; maximum and minimum frequencies were measured 20dB below the peak frequency to avoid background noise interference. Call description follows the call centered approach of Köhler et al. [32]. As the call is produced by multiple expiratory movements, with 100% amplitude modulation between the emission and the silence interval, we chose to treat it as multiple notes call sensu Köhler et al. [32], not as a pulsed single note call (sensu Brown et al. [4]). We consider that all *Ranitomeya* species may have this call structure, thus pulses sensu Brown et al. [4] will be interpreted here as notes. Spectrogram and oscillogram were generated in R environment [33] through the ‘*seewave*’ package 2.0.5 [34] using a Hanning window, 256 points of resolution (Fast Fourier Transform) and an overlap of 85%. Bioacoustic raw data is provided in S2 Table.

### Tadpole description

The external morphology of *Ranitomeya* sp. nov. tadpole was described based on five individuals: one at stage 26, one at stage 30, two at stage 37, and one at stage 38 [35]. Thirty-seven morphometric measurements of the five tadpoles were taken to the nearest 0.01 mm using a Leica stereomicroscope (model S8APO) coupled to a Leica DFC295 camera.

Morphometric measurements taken for tadpoles follow Randrianiaina et al. [36]: total length (**TL**), body length (**BL**), tail length (**TAL**), maximum body height (**BH**), maximum body width (**BW**), body height on the nostril (**BHN**), body height on the eyes (**BHE**), body width on the nostril (**BWN**), body width on the eyes (**BWE**), tail muscle width at base (**TMW**), maximum tail height (**MTH**), dorsal fin height (**DF**), ventral fin height (**VF**), tail muscle height (**TMH**), interorbital distance (**IOD**), internarial distance (**IND**), rostro-eye distance (**RED**), rostro-nostril distance (**RND**), rostro-spiracle distance (**RSD**), eye diameter (**ED**), eye-nostril distance (**END**), spiracle length (**SL**), spiracle width (**SW**), spiracle height (**SH**), vent tube length (**VL**), oral disc width (**ODW**), anterior (upper) labium (**AL**), posterior (lower) labium (**PL**), first anterior tooth row (**A1**), second anterior tooth row (**A2**), medial gap in second anterior tooth row (**A2 GAP**), first posterior tooth row (**P1**), second posterior tooth row (**P2**), third posterior tooth row (**P3**), medial gap in the first posterior tooth row (**P1 GAP**), upper jaw sheath width (**UJW**) and, finally, upper jaw sheath length (**UJL**).

### Sequence obtention and genetic analysis

Genomic DNA was extracted from nine adults and three larvae (liver, muscle or tail tissues) from type locality (S3 Table). We extracted genomic DNA using PureLink™ Genomic DNA (Invitrogen by Thermo Fisher Scientific, Carlsbad, CA, USA). Sequences of four mitochondrial loci (12S rRNA, 16S rRNA, Cytochrome C Oxidase sub-unit 1 – CO1 and Cytochrome B – CYTB) were amplified by polymerase chain reaction (PCR) with general final volume of 15 μL containing 1.5 μL of 25 mM MgCl2, 1.5 μL of 10 mM dNTPs (2.5 mM each dNTP), 1.5 μL of tampon 10× (75 mM Tris HCl, 50 mM KCl, 20 mM (NH4)2SO4), 1,5 μL of forward primer (2 μM)), 1,5 μL of reverse primer (2 μM), 6.4 μL of ddH2O and 0.1 μL of 1 U Taq DNA Polymerase and 1 μL of DNA (30–50 ng/μL). For 12S we used primers 12S L13 (5’-TTAGAAGAGGCAAGTCGTAACATGGTA-3’) [37] and 12S Titus I (5’-GGTGGCTGCTTTTAGGCC-3’) [38] with the following PCR program: 90 s at 94°C followed by 35 cycles of 94°C (45 s), 55°C (45 s) and 72°C (90 s), and final extension of 7 minutes at 72°C. For 16S we used primers 16Saf (5’-CGCCTGTTTATCAAAAACAT-3’) and 16Sbr (5’-CCGGTCTGAACTCAGATCACGT-3’) [39] with the following PCR program: 90 s at 94°C followed by 35 cycles of 94°C (45 s), 55°C (45 s) and 72°C (90 s), and final extension of 7 minutes at 72°C. For COI we used primers Chmf4f (5’-TYTCWACWAAYCAYAAAGAYATCGG-3’) and Chmr4r (5’-ACYTCRGGRTGRCCRAARAATCA-3’) [40] with the following PCR program: 60 s at 94°C followed by 35 cycles of 94°C (20 s), 50°C (50 s) and 72°C (90 s), and final extension of 10 minutes at 72°C. Finally, for CYTB we used primers MVZ 15-L (5’-GAACTAATGGCCCACACWWTACGNAA-3’) [41] and H15149 (5’-AAACTGCAGCCCCTCAGAAATGATATTTGTCCTCA-3’) [42] with the following PCR program: 120 s at 95°C followed by 35 cycles of 95°C (30 s), 45°C (60 s) and 72°C (90 s), and final extension of 6 minutes at 72°C.

All PCR products were visualized in 1% agarose with SYBRSafe (Life Inc.) and purified using PEG 8000 protocol [43] and submitted to sequencing using standard protocols of the Big DyeTM Terminator Kit (Applied Biosystems, Inc., Grand Island, NY, USA). Forward and reverse amplicons were sequenced in an ABI PRISMI 3500XL (Thermo Fisher). Sequence quality was checked by accessing the chromatogram, the consensus sequences were generated and manually edited using the Geneious Pro 5.4.6 (Biomatters Ltd.). Sequences were subjected to BLAST searches [44] in GenBank to verify if the target had been amplified and its quality was manually checked. The consensus sequences of each specimen were deposited in GenBank (S3 Table). To infer the phylogenetic relationships of the new species, a data set containing homologous sequences was retrieved from GenBank (S3 Table). We selected sequences that represent all the diversity of *Ranitomeya* species, preferably containing material assigned to the type series or from type locality as well as four sequences from representatives of the two closest genera, *Andinobates* and *Excidobates*. Our complete dataset comprises 266 sequences of the four loci (33 for 12S, 120 for 16S, 17 for CO1 and 96 for CYTB) that correspond to 121 terminals. Sequences of each locus were aligned using MAFFT online server using E-INS-i strategy for 12S and 16S gene and G-INS-i for CO1 and CYTB [45]. The final matrix was composed of 121 terminals with 2,419 bp (632 pb for 12s, 532 pb for 16S, 656 pb for COI, and 599 pb for CYTB).

### Delimitation analysis and genetic distances

The Operational Taxonomic Units (OTUs) were delimited using three DNA-based species delimitation methods: (1) the pairwise distance-based method Assemble Species by Automatic Partitioning (ASAP, [46]) (2) the Bayesian implementation of the Poisson Tree Processes model approach (bPTP; [47]); and (3) the Generalized Mixed Yule Coalescent method (single threshold GMYC; [48,49]). All methods were performed with the 16S locus, and additionally ASAP was performed with the 12S and CYTB loci to confirm the delimitation of the new species. We defined Operational Taxonomic Units (OTUs) by the majority-rule consensus of the three partitions obtained with 16S locus (i.e., a lineage is considered as an OTU when it appeared in at least two out of the three results). The pairwise interspecific and the intraspecific genetic distances using pairwise deletion were calculated between the populations of new species and close relatives using MEGA 11 [50] for 16S (p-distance and Kimura-two-parameter, [51]), 12S and CYTB loci (p-distance).

### Phylogenetic tree reconstruction

Phylogenetic analyses were performed with Bayesian Inference (BI) using the complete matrix for the four loci on the software Beast 2.7.1 [52]. Coding loci were partitioned to analyze each codon position independently. Two independent runs on 5x10^7^ generations of the MCMC were carried, sampled every 5000 generations. The best nucleotide substitution model was selected using bModelTest [53] using the “named extended models” parameters in the MCMC [53]. The best-fitting substitution model for each partition was evaluated with BModelAnalyser package. The chosen models were GTR for 12S, 16S, second position of COI and third position of CYTB; TN93 for the first and third positions of COI, second position of CYTB; and finally, TVM for the first position of CYTB. The clock was set to a strict clock model to estimate the evolutionary rates, as the branches closest to the new species may have homogeneous rates. The tree prior used was Yule, as significant extinction events were not expected in this group, other priors were set to default. A post-burnin of 10% was used to remove the instability of the initial chain. We assessed MCMC convergence using the software Tracer 1.7.2 [54] considering ESS values higher than 300. The maximum clade credibility tree was annotated with the software TreeAnnotator v2.5.0 [55] and visualized in the software FigTree [56].

### Nomenclatural acts

The electronic edition of this article conforms to the requirements of the amended International Code of Zoological Nomenclature, and hence the new names contained herein are available under that Code from the electronic edition of this article. This published work and the nomenclatural acts it contains have been registered in ZooBank, the online registration system for the ICZN. The ZooBank LSIDs (Life Science Identifiers) can be resolved and the associated information viewed through any standard web browser by appending the LSID to the prefix “http://zoobank.org/”. The LSID for this publication is: urn:lsid:zoobank.org:pub:585AABCE-B0A5-4684-8C23-2792498DFF7D. The electronic edition of this work was published in a journal with an ISSN and has been archived and is available from the following digital repositories: LOCKSS.

### Animal ethics

Protocols of collection and animal care follow the Brazilian Federal Council for Biology (Resolution number 148/2012) and the Ethics Committee on the Use of Animals of the Instituto Nacional de Pesquisas da Amazônia - CEUA-INPA (Process n° 35/2020, SEI 01280.001134/2020–63).

### Field study permissions

The Centro Nacional de Pesquisa e Conservação de Répteis e Anfíbios of the Instituto Chico Mendes de Conservação da Biodiversidade – ICMBio (Ministry of Environment, Government of Brazil) approved the field permit (permit number 13777–1).

## Results

### Phylogenetic relationships and genetic distances

**Fig 1:**
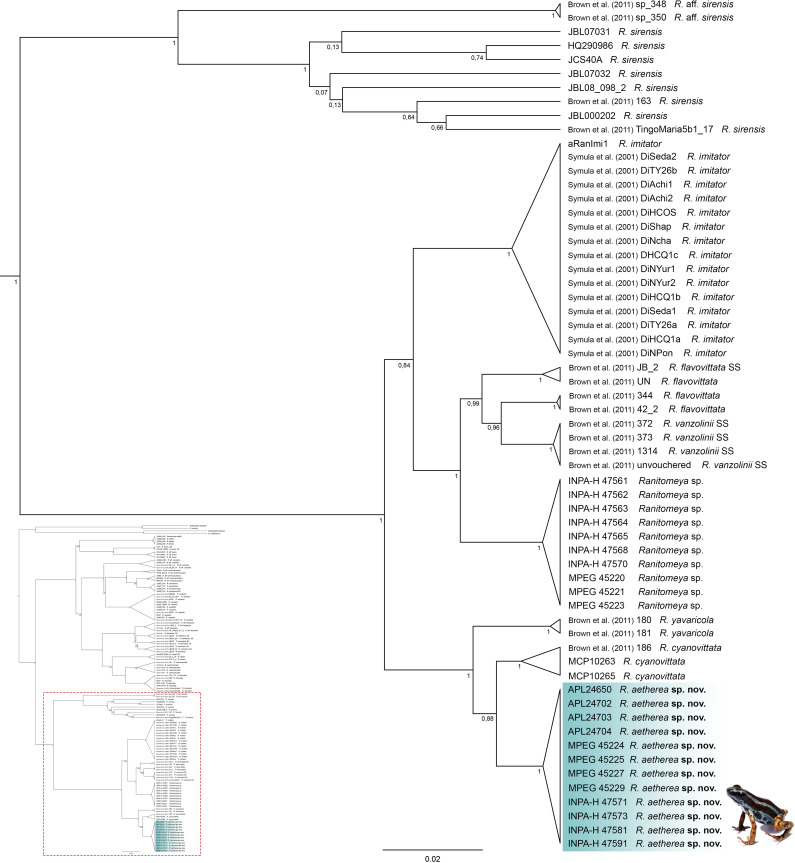
Part of the *Ranitomeya* genus phylogenetic tree depicting the relationships of *Ranitomeya aetherea* sp. nov. with the other species of *R. vanzolinii* species group (Bayesian inference tree for genes 16S, 12S, COI and CytB). Posterior probability support is shown on the branches. *SS*: *Strictu Sensu* – individual in the type series. The species’ name is preceded by the specimen voucher number (for the complete tree see S2 Fig).

The new species was confirmed in all delimitation methods (S4 Table) and its individuals show low intraspecific uncorrected genetic distance (16S p-distance: mean = 0.13%, maximum = 0.24%). The new species is nested within the *Ranitomeya vanzolinii* species group sensu Brown et al. [4]. It is sister species to *R. cyanovittata* Pérez-Peña et al. [12] and together with it forms a strongly supported clade (posterior probability = 1) with *R. yavaricola* Pérez-Peña et al. [12] (Fig 1, S2 Fig). The clade composed of these three species has 16S interspecific uncorrected p-distances ranging from 2.04% to 4.53% (Table 1), 12S interspecific uncorrected p-distances ranging from 2.05% to 3.25% (S5 Table), CYTB interspecific uncorrected p-distances ranging from 3.95% to 4.40% (S6 Table). All these species occur in western Amazonia.

**Table 1:** Interspecific and intraspecific genetic distances (16S) between *Ranitomeya aetherea* sp. nov. and closely related taxa.

Species	1	2	3	4	5	6	7	8	9	10
1. *R. aetherea* **sp. nov.**	**0.13**	3.13	2.08	3.13	2.26	3.16	8.16	8.00	2.55	3.27
2. *Ranitomeya* sp.	3.05	**0.00**	4.03	4.05	3.16	3.02	9.97	10.0	3.45	4.74
3. *R. cyanovittata*	2.04	3.89	**0.97**	3.96	3.76	3.86	9.14	8.99	4.06	3.63
4. *R.* aff. *flavovittata*	3.06	3.91	4.00	**0.00**	1.62	4.37	10.4	10.5	2.05	5.53
5. *R. flavovittata*	2.21	3.07	3.62	1.60	**0.25**	3.73	9.48	9.64	1.68	4.90
6. *R. imitator*	3.07	2.94	3.73	4.21	3.60	**0.60**	8.85	9.29	4.01	3.97
7. *R. sirensis*	7.67	9.19	8.51	9.61	8.78	8.26	**1.65**	5.65	9.25	6.93
8. *R.* aff. *sirensis*	7.56	9.27	8.43	9.78	8.97	8.68	5.40	**0.00**	9.91	8.01
9. *R. vanzolinii*	2.50	3.35	3.92	2.02	1.66	3.87	8.61	9.21	**0.12**	4.65
10. *R. yavaricola*	3.19	4.53	3.52	5.28	4.69	3.84	6.58	7.56	4.47	**0.24**

Uncorrected p-distances (%; lower diagonal) and Kimura-2-parameter (%; upper diagonal) for sequences in a matrix with 532 characters from 16S mtDNA gene and expressed as percentages. Numbers in bold represent intraspecific p-distance values.

## Taxonomic account

*Ranitomeya* *aetherea* **sp. nov.** urn:lsid:zoobank.org:act:5327336E-8444-479E-83FE-3B3018919C4E

Figs 2–6, 9 and 11.

**Fig 2:**
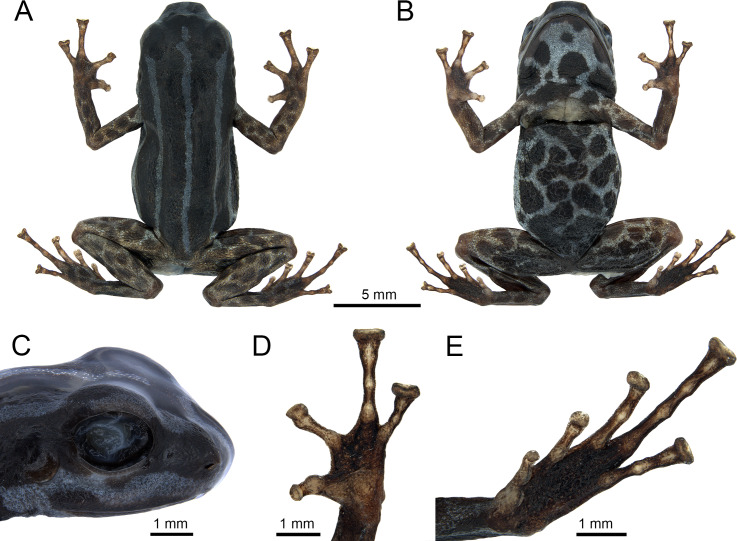
Preserved holotype of *Ranitomeya aetherea* sp. nov. (INPA-H 47581) from Comunidade de Nova Esperança, Eirunepé municipality, Amazonas state, Brazil: (A) dorsal and (B) ventral view, (C) lateral head, (D) hand and (E) foot. Photographs: A. T. Mônico.

**Fig 3:**
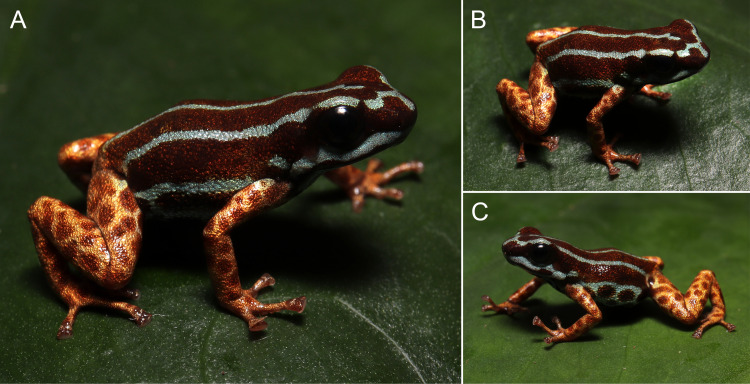
In life holotype of *Ranitomeya aetherea* sp. nov. (INPA-H 47581, APL 24826): (A) lateral and (B) dorsal view, (C) lateral view showing the spots in lateroventral and inguinal region. Photographs: A. T. Mônico.

**Fig 4:**
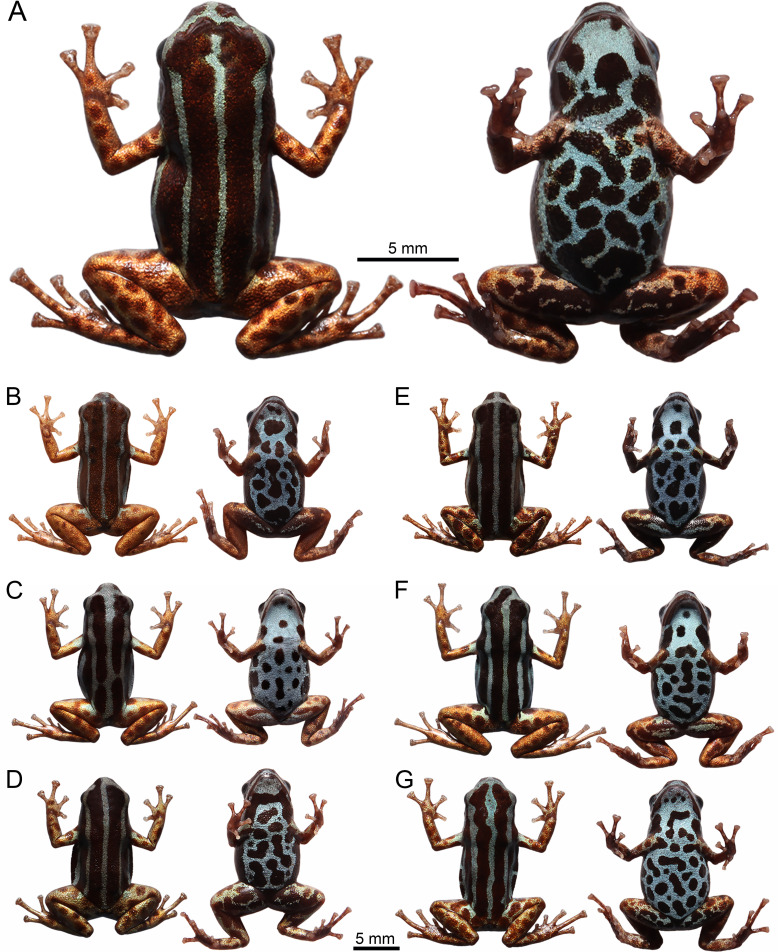
Dorsal and ventral color pattern variation of the *Ranitomeya aetherea* sp. nov. in life: Males (A) Holotype - INPA-H 47581, (B) INPA-H 47575, (C) INPA-H 47584, (D) INPA-H 47588; females (E) INPA-H 47580, (F) INPA-H 47583, (G) INPA-H 47584. Photographs: A.T. Mônico.

**Fig 5:**
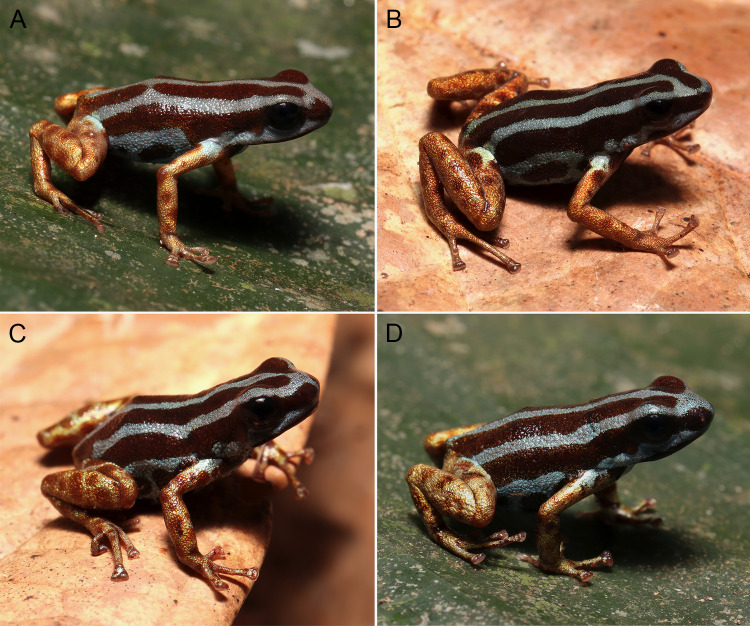
Adult individuals of *Ranitomeya aetherea* sp. nov.: [A] paratype, male INPA-H 47584, APL 24839; [B] paratype, female INPA-H 47583, APL 24828; [C] paratype, female INPA-H 47584, APL 24829; and [D] paratype, male INPA-H 47587, APL 24840. Photographs: A.T. Mônico.

**Fig 6:**
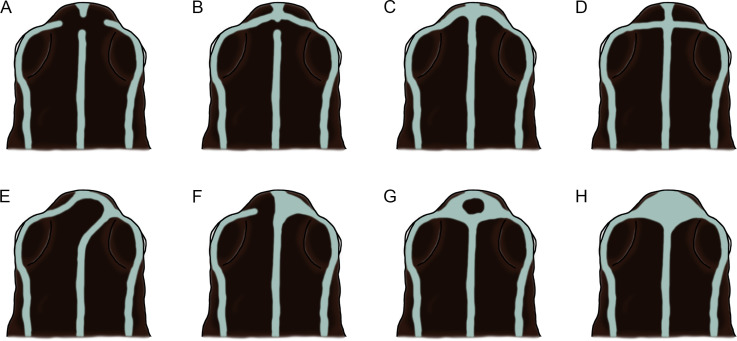
Schematic illustration of the variation of stripes on the head of the adult individuals of *Ranitomeya aetherea* sp. nov.

**Fig 7:**
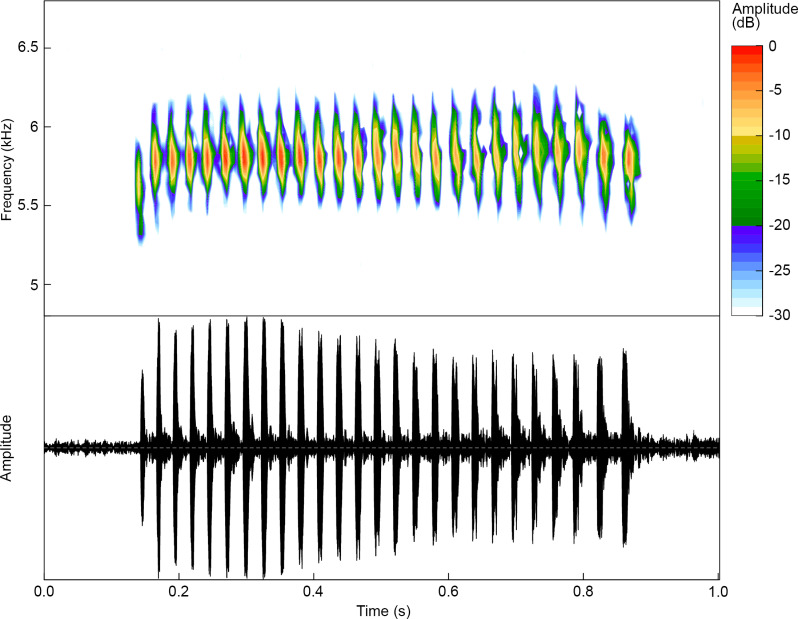
Advertisement call of a paratype of *Ranitomeya aetherea* sp. nov. (INPA-H 47573, FNJV 0124341, SVL = 15.6 mm) recorded at the Comunidade de Nova Esperança, Eirunepé municipality, Amazonas state, Brazil. Air temperature 27.3° C.

**Fig 8:**
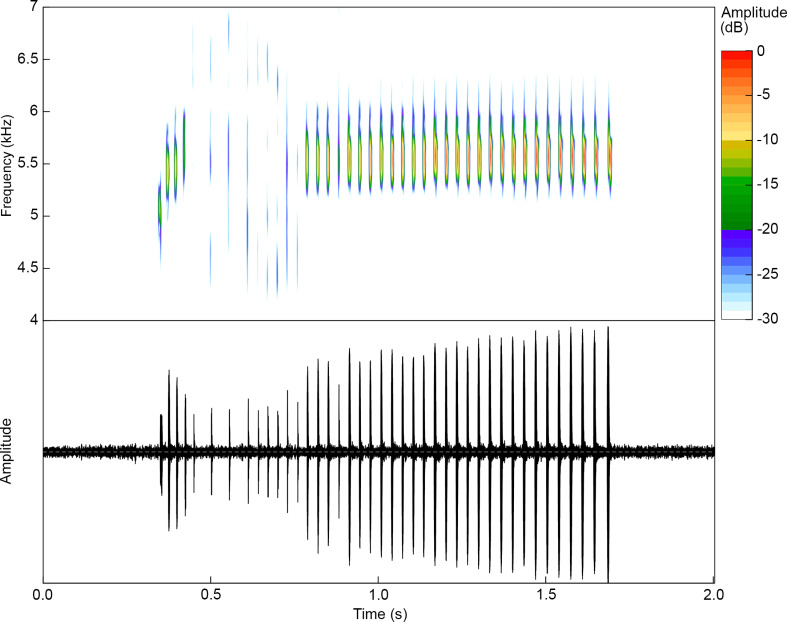
Courtship call of a paratype of *Ranitomeya aetherea* sp. nov. (MPEG 45225, FNJV 0124340, 15.8 mm). Air temperature 24.5°C.

**Fig 9:**
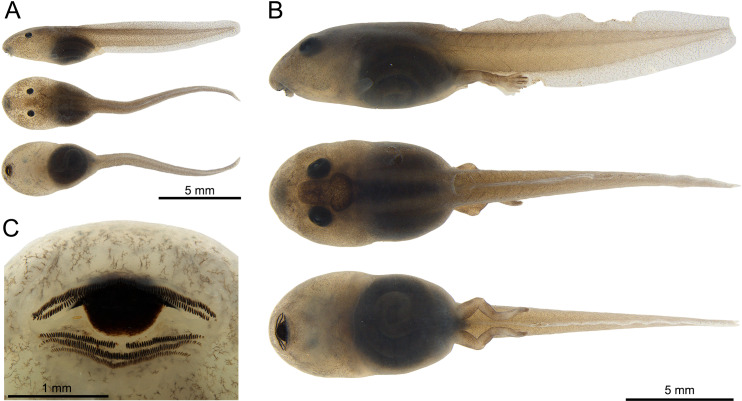
Preserved tadpoles of *Ranitomeya aetherea* sp. nov.: (A) stage 26, (B) stage 37 (in lateral, dorsal and ventral views), (C) ventral view of the oral disc at stage 37. Photographs: A.T. Mônico.

**Fig 10:**
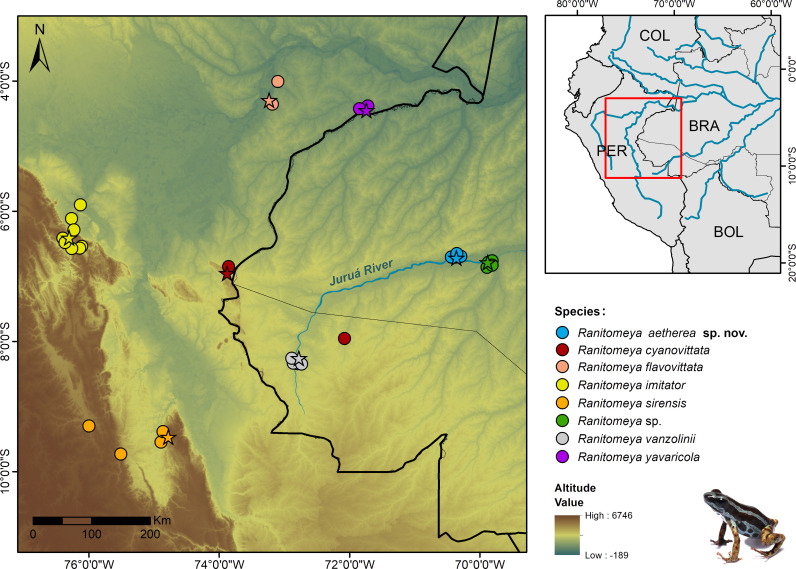
Geographic distribution of the *Ranitomeya aetherea* sp. nov. and species of *R. vanzolinii* group. Stars indicate the type localities of each species. Acronyms: BRA, Brazil; PER, Peru; COL, Colombia; BOL, Bolivia. All shapefiles are under open access license and free to use, credited to: USGS (Elevation layer, GTOPO30, https://earthexplorer.usgs.gov/), ANA (Brazilian States, South America political boundaries and Hydrography, https://metadados.snirh.gov.br/geonetwork/srv/por/catalog.search#/home).

**Fig 11:**
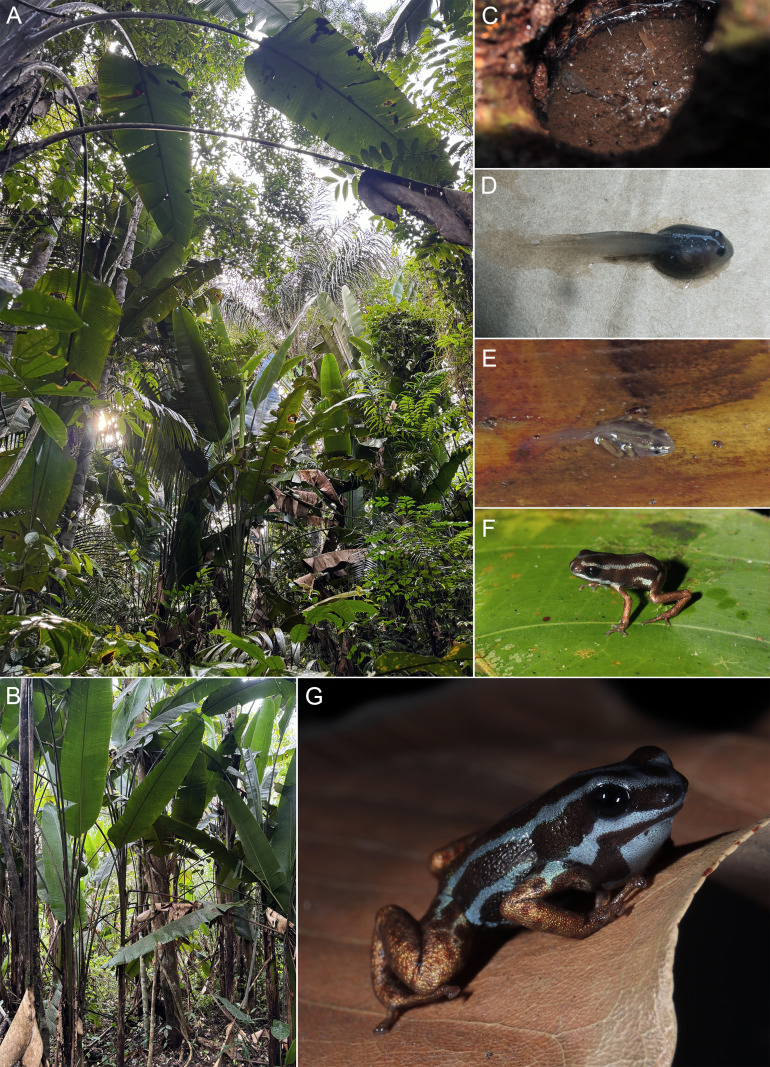
*Ranitomeya aetherea* sp. nov. natural history and breeding aspects: [A] ombrophilous open forests with palms inhabited by the new species; [B] habitat of the new species in detail; [C] single tadpole within a phytotelma; [D] tadpole with a partly developed dorsal pattern of the adult individuals; [E] metamorph displaying adult coloration; [F] juvenile; [G] calling male with inflated vocal sac. Photographs: A.T. Mônico (A, B, C, F and G), A.P. Lima (D), S. Dantas (E).

*Ranitomeya* cf. *yavaricola* – Twomney et al. [57]

*Ranitomeya cyanovittata* – Lima et al. [11]

*Ranitomeya* aff. *cyanovittata* – Mônico et al. [58]

### Holotype

INPA-H 47581 (field number APL 24826; Fig 2), adult male collected at the RAPELD sampling module of the Comunidade de Nova Esperança, Eirunepé municipality, Amazonas state, Brazil (6°42’12.7”S, 70°22’12.1”W; 135 m a.s.l.) on 19 March 2024 by Alexander Tamanini Mônico and Albertina Pimentel Lima.

### Paratypes

Twenty-five adult specimens (18 males and seven females), all collected at the same locality as the holotype: six males [MPEG 45225, MPEG 45226, MPEG 45227, INPA-H 47571, INPA-H 47573, INPA-H 47574] and one female [MPEG 45224] collected on 20 March 2023 by A.P. Lima, A. Ferreira, J. Dayrell and S. Dantas; one male [INPA-H 47572] collected on 21 March 2023 by A.P. Lima, A. Ferreira, J. Dayrell and S. Dantas; six males [INPA-H 47575, INPA-H 47576, INPA-H 47577, INPA-H 47578, INPA-H 47579, INPA-H 47582] and three females [INPA-H 47580, INPA-H 47583, INPA-H 47584] collected on 19 March 2024 by A.T. Mônico and A.P. Lima; five males [INPA-H 47586, INPA-H 47587, INPA-H 47588, INPA-H 47589, MPEG 45229] and three females [INPA-H 47590, MPEG 45228, INPA-H 47591] collected on 20 March 2024 by A.T. Mônico and A.P. Lima.

### Etymology

The specific epithet *aetherea* is a Latin adjective meaning “heavenly”, which philosophically refers to the coloration of the species’ dorsal stripes: a shade of blue reminiscent of the sky. In addition, we attribute this name to one’s feeling of enchantment and delicacy when encountering these frogs, as if they were from outside this world.

### Generic placement

We assign the new species to *Ranitomeya*, based on the phylogenetic placement (Fig 1) and the following external characteristics: coloration is bright and aposematic, finger I is greatly reduced and shorter than finger II, finger discs two and four are greatly expanded, dorsal skin texture is smooth (to shagreen), toe webbing is absent (see Brown et al. [4]; Kahn et al. [2]).

### Characterization

The new species is characterized by the following characters: (1) dorsum dark reddish-brown (brick red, color 36 by Köhler [30]) with three parallel light blue stripes (light-sky blue, color 191 by Köhler [30]), middorsal stripe extending from the tip of snout to slightly before the vent (always complete from between the eyes to the vent), dorsolateral stripes always complete extending from the snout to the groin; (2) limbs copper metallic (light orange yellow, color 7 by Köhler [30]) with light reddish-brown (pratt’s rufous, color 72 by Köhler [30]) spots and irregular light-sky blue spots at their bases; (3) venter and gular region light blue (cyan white, color 155 by Köhler [30]) with dark reddish-brown spots (vandyke brown, color 282 by Köhler [30]); (4) dorsal skin smooth to shagreen; (5) gular skin smooth to shagreen; belly skin shagreen to weakly granular in the center, granular between the arms, near to the cloaca and to the flanks, forming a ring shaped granular region; (6) skin on the limbs shagreen, posteroventral surface of thighs granular; (7) SVL in adult males 15.2–17.0 mm (n = 19), in adult females 14.4–16.9 mm (n = 7); (8) head slightly wider than long (HW/WL = 1.12–1.35),; (9) HL 25–29% of SVL; (10) snout truncate in ventral, dorsal and lateral view; (11) *canthus rostralis* rounded, loreal region flat; (12) nostril directed laterally at the angle of the snout, internarial distance 35–40% of head width; (13) tympanic membrane and annulus visible, rounded, 32–52% of ED, posterodorsal margin of tympanum hidden by the mandibular depressor muscles; (14) tongue ovoid, attached anteriorly; (15) vocal slits present in males, lying laterally under the tongue in the posterior region of the mouth floor; (16) dentigerous processes of vomers absent; (17) choanae small, ovoid; (18) HL 24–29% of SVL, AL 27–32% of SVL; (19) relative length of fingers III > IV > II > I, L1F 55–69% of L2F, finger discs slightly expanded and rounded on Finger I, greatly expanded and truncated on fingers III and IV; (20) thenar and palmar tubercles diffuse, ovoid; (21) proximal subarticular tubercles present on each finger, distal subarticular tubercles present on fingers III–IV; (22) KK 80–93% of SVL, TL 86–99% of FL; (23) relative length of toes IV > III > V > II > I, toes with poorly developed lateral fringes, L1T 48–64% of L2T, finger discs not expanded and rounded on Finger I, highly expanded and truncated on toes IV and V; (24) outer metatarsal tubercle ovoid, inconspicuous, inner metatarsal tubercle ovoid; (25) proximal subarticular tubercles present at the base of each toe, distal subarticular tubercles present on toes III and V, one medial subarticular tubercle on Toe IV; (26) advertisement call resembling a cricket-like long-lasting trill consists of 16–35 tonal notes and it is characterized by average CD = 760 ± 120 ms, SBC = 4.7–33.6 s, ND = 11.3 ± 1.38 ms, SBN = 18.8 ± 2.91 ms, NR = 33.6 ± 2.2 notes/s, and DF = 5,168–6,029 Hz; (27) tadpole TAL 60% of TL, tadpole TMW 50–54% of MTH; (28) in tadpole stages 31–38 labial tooth row formula 2(2)/3(1) and tooth rows P1 = P2 > P3, P3 from 83–87% of P1; (29) in life, tadpole tail musculature light red-brown, dorsal and ventral fins transparent with light red-brown tiny reticules, final tadpole stages 37–38 with light sky-blue dorsal stripes.

### Description of holotype

INPA-H 47581 An adult male (Figs 2 and 3), SVL 15.7 mm, body moderately robust, head slightly wider than long (HW/HL = 1.35), HL = 35.7% of SVL. Snout truncate in dorsal, lateral and ventral view. Nostril directed laterally at the angle of the snout, TSCN = 1.1 mm from the tip of the snout, IND = 2.0 mm, 36.0% of HW*. Canthus rostralis* rounded, loreal region flat. END = 1.54 mm, 72.4% of ED. Tympanic annulus and tympanic membranae present, tympanum rounded, posterodorsal margin hidden by depressor muscle, TD 46.8% of ED. Tongue ovoid, attached anteriorly, Dentigerous processes of vomers absent. Choanae ovoid and small (0.6 mm), positioned far laterally, concealed by palatal shelf of maxilla, not visible in ventral view. Vocal slits are present, located in the posterior region of the mouth floor, laterally under the tongue, easily distinguishable.

Forelimbs slender, hand relatively large (HaL 25.3% of SVL). Finger I considerably shorter than Finger II (L1F 54.7% of LF2); fingers III > IV > II > I. Discs on fingers III, IV greatly expanded, Finger II moderately expanded truncate, disc of Finger I slightly expanded, rounded. Hands lacking lateral fringes and webbing. Ulnar tubercles absent. Palmar and thenar tubercles diffuse and ovoid. Proximal subarticular tubercles present in each finger, poorly pigmented. Proximal subarticular tubercle of Finger I two times greater than others, and of Finger IV diffuse. Poorly pigmented distal subarticular tubercles present in fingers III–IV (Fig 2).

Length of legs moderate; Femur and tibia nearly equal in length; TL 97.1% of FL; KK 84.8% of SVL. Relative lengths of appressed toes IV > III > V > II > I. Toe I short, with disc rounded not expanded; Toe II with slightly expanded, rounded disc; toes III–V with highly expanded, truncated discs; toes with poorly developed lateral fringes. Tarsal tubercle absent, feet lacking webbing. Outer metatarsal tubercle present, ovoid, but almost indistinguishable. Inner metatarsal tubercle ovoid, poorly pigmented. Subarticular tubercles present at base of each toe, but most visible on toes I, II, III and V, due poorly pigmentation, diffused in Toe IV. Distal subarticular tubercles present on Toe III and V, poorly distinguishable on Toe IV. One medial subarticular tubercle diffused in Toe IV (Fig 2). All measurements of the holotype are given in Table 2.

**Table 2:** Morphometric measurements of adult type specimens of *Ranitomeya aetherea* sp. nov.

Morphometric measurements (mm)	Holotype	Males (N = 18)	Females (N = 7)
SVL – Snout-vent length	15.7	15.9 ± 0.44 (15.2–17.0)	16.1 ± 0.82 (14.4–16.9)
HL – Head length	4.2	4.3 ± 0.22 (4.1–4.8)	4.3 ± 0.24 (3.9–4.6)
HW – Head width	5.6	5.3 ± 0.16 (5.0–5.7)	5.3 ± 0.19 (4.9–5.6)
IOD – Interorbital distance	1.8	1.9 ± 0.13 (1.6–2.2)	1.9 ± 0.19 (1.6–2.2)
UEW – Upper eyelid width	1.4	1.3 ± 0.10 (1.1–1.4)	1.3 ± 0.08 (1.1–1.3)
MTD – Mouth-tympanum distance	0.6	0.6 ± 0.07 (0.4–0.7)	0.6 ± 0.08 (0.5–0.7)
TD – Tympanum diameter	1.0	0.9 ± 0.13 (0.7–1.2)	0.9 ± 0.10 (0.7–1.0)
DET – Distance from eye to tympanum	0.5	0.6 ± 0.05 (0.5–0.6)	0.5 ± 0.06 (0.5–0.6)
ED – Eye diameter	2.1	2.0 ± 0.08 (1.9–2.2)	2.0 ± 0.11 (1.8–2.2)
SL – Snout length	2.1	1.8 ± 0.14 (1.6–2.2)	1.8 ± 0.20 (1.4–2.0)
END – Eye-nostril distance	1.5	1.4 ± 0.09 (1.2–1.5)	1.4 ± 0.16 (1.0–1.5)
BW – Body width	5.4	5.2 ± 0.23 (4.6–5.7)	5.5 ± 0.41 (4.9–6.1)
TSCN – Snout-nostril distance	1.1	1.0 ± 0.07 (0.9–1.2)	1.1 ± 0.06 (1.0–1.1)
IND – Internarial distance	2.0	2.0 ± 0.08 (1.8–2.2)	2.0 ± 0.10 (1.9–2.1)
AL – Arm length	4.4	4.5 ± 0.21 (4.1–4.8)	4.7 ± 0.31 (4.1–5.2)
FAL – Forearm length	3.8	3.8 ± 0.16 (3.4–4.0)	3.9 ± 0.22 (3.5–4.2)
HaL – Hand length	4.0	4.1 ± 0.16 (3.8–4.5)	4.3 ± 0.35 (3.7–4.9)
L1F – Finger I length	1.6	1.7 ± 0.09 (1.6–2.0)	1.8 ± 0.14 (1.5–2.0)
L2F – Finger II length	2.9	2.8 ± 0.16 (2.5–3.0)	2.9 ± 0.15 (2.6–3.1)
L3F – Finger III length	4.0	4.1 ± 0.16 (3.8–4.5)	4.3 ± 0.35 (3.7–4.9)
L4F – Finger IV length	3.1	3.2 ± 0.15 (2.9–3.5)	3.2 ± 0.26 (2.7–3.6)
W1FD – Width of disc on finger I	0.5	0.4 ± 0.05 (0.3–0.5)	0.4 ± 0.06 (0.3–0.5)
W1F – Width of finger I just below disc	0.4	0.4 ± 0.04 (0.3–0.4)	0.4 ± 0.06 (0.3–0.4)
W2FD – Width of disc on finger II	0.6	0.6 ± 0.06 (0.5–0.7)	0.6 ± 0.07 (0.5–0.7)
W2F – Width of finger II just below disc	0.5	0.5 ± 0.05 (0.3–0.5)	0.5 ± 0.07 (0.4–0.6)
W3FD – Width of disc on finger III	0.9	0.9 ± 0.10 (0.6–1.1)	0.9 ± 0.09 (0.8–1.0)
W3F – Width of finger III just below disc	0.7	0.7 ± 0.07 (0.5–0.8)	0.7 ± 0.08 (0.6–0.8)
W4FD – Width of disc on finger IV	0.8	0.8 ± 0.10 (0.5–1.0)	0.8 ± 0.10 (0.6–0.9)
W4F – Width of finger IV just below disc	0.6	0.6 ± 0.07 (0.4–0.7)	0.6 ± 0.08 (0.4–0.7)
KK – Knee-knee distance	13.3	13.5 ± 0.43 (12.3–14.4)	13.7 ± 0.29 (12.4–15.3)
FL – Femur length	6.6	6.7 ± 0.21 (6.2–7.2)	6.8 ± 0.33 (6.1–7.2)
TL – Tibia length	6.4	6.4 ± 0.22 (5.7–6.7)	6.3 ± 0.44 (5.4–7.0)
TaL – Tarsus length	4.0	4.0 ± 0.25 (3.3–4.4)	4.0 ± 0.29 (3.5–4.6)
FoL – Foot length	5.9	5.9 ± 0.28 (5.3–6.5)	6.1 ± 0.47 (5.4–7.1)
L1T – Toe I length	1.5	1.5 ± 0.13 (1.2–1.8)	1.6 ± 0.20 (1.2–1.9)
L2T – Toe II length	2.7	2.7 ± 0.15 (2.4–3.1)	2.8 ± 0.30 (2.3–3.3)
L3T – Toe III length	4.2	4.4 ± 0.19 (4.1–4.9)	4.6 ± 0.31 (4.0–5.1)
L4T – Toe IV length	5.9	5.9 ± 0.28 (5.3–6.5)	6.1 ± 0.47 (5.4–7.1)
L5T – Toe V length	4.0	4.0 ± 0.21 (3.6–4.4)	4.1 ± 0.35 (3.7–4.8)
W1TD – Width of disc on toe I	0.3	0.4 ± 0.04 (0.3–0.4)	0.4 ± 0.05 (0.3–0.4)
W1T – Width of toe I just below disc	0.3	0.3 ± 0.04 (0.3–0.4)	0.3 ± 0.05 (0.3–0.4)
W2TD – Width of disc on toe II	0.5	0.5 ± 0.05 (0.4–0.6)	0.5 ± 0.04 (0.4–0.5)
W2T – Width of toe II just below disc	0.5	0.4 ± 0.04 (0.3–0.5)	0.4 ± 0.03 (0.3–0.4)
W3TD – Width of disc on toe III	0.6	0.6 ± 0.06 (0.5–0.8)	0.6 ± 0.07 (0.5–0.7)
W3T – Width of toe III just below disc	0.5	0.5 ± 0.05 (0.4–0.6)	0.5 ± 0.04 (0.4–0.6)
W4TD – Width of disc on toe IV	0.8	0.8 ± 0.11 (0.6–1.1)	0.8 ± 0.06 (0.7–0.8)
W4T – Width of toe IV just below disc	0.7	0.6 ± 0.07 (0.5–0.8)	0.6 ± 0.06 (0.5–0.7)
W5TD – Width of disc on toe V	0.7	0.7 ± 0.08 (0.5–09)	0.7 ± 0.09 (0.6–0.8)
W5T – Width of toe V just below disc	0.6	0.6 ± 0.06 (0.4–0.7)	0.6 ± 0.04 (0.5–0.6)

Values express mean ± standard deviation and range.

Skin texture smooth to shagreen on head and most of dorsum. Ventral surface of hindlimbs shagreen, posterior surface of thighs granular. Gular region smooth to shagreen, belly skin shagreen to weakly granular in the center, granular between the arms, near to the cloaca and to the flanks, forming a ring shaped granular region. Arms shagreen.

In life, dorsum reddish-brown (brick red, color 36 by Köhler [30]) with three light blue parallel stripes (light-sky blue, color 191 by Köhler [30]) (Figs 3 and 4), middorsal stripe extends from the tip of snout to slightly before the vent, with a break anterior to eyes. Dorsolateral stripes extend from the snout, where they fuse with the middorsal stripe, to the groin with its color leaking slightly on the thighs forming irregular spots. Venter and gular region light blue (cyan white, color 155 by Köhler [30]) with dark reddish-brown spots (vandyke brown, color 282 by Köhler [30]). Both forelimbs and hindlimbs metallic copper (light orange yellow, color 7 by Köhler [30]) with light reddish-brown spots (pratt’s rufous, color 72 by Köhler [30]), limb and ventral patterns gradually merging in the ventral surface of the thighs proximal to the body. Iris black.

After four months in alcohol, general color pattern remained, but colors faded, light color surfaces with dense dark melanophores (Fig 2).

### Variation in the type series

Snout-vent length of the type series ranges from 15.2 to 17.0 mm in males (n = 19) and from 14.4 to 16.9 mm in females (n = 7) (Table 2).

The pattern of stripes on the dorsum is very constant (Figs 4 and 5), two individuals (MPEG 45227 and INPA-H 47591) had a small break on middorsal stripe, and four (INPA-H 47573, 47574, 47589 and 47591) had a short connection between the middorsal and one dorsolateral stripe on the dorsum.

The stripes on the head show a strong tendency to fuse in the snout region (Fig 6). The type specimens display the following patterns: (1) none of the stripes connected, middorsal stripe with a gap anterior to the eyes (n = 1; Fig 6A), (2) middorsal stripe with a short interruption anterior to the eyes, dorsolateral stripes connected with the middorsal stripe on the tip of snout (n = 4; Fig 6B), (3) middorsal stripe complete, all dorsal stripes connected on the tip of snout (n = 2; Fig 6C), (4) middorsal stripe complete, all dorsal stripes connected slightly anterior to the eyes (n = 4; Fig 6D), (5) middorsal stripe connected laterally with one of the dorsolateral stripes, dorsolateral stripes connected on the tip of snout (n = 4; Fig 6E), (6) middorsal stripe complete, one of the dorsolateral stripes fused with the middorsal stripe on the side of snout anterior to the eyes (n = 1; Fig 6F), (7) all stripes fused on the tip of snout leaving a spot of ground dorsal coloration in the middle of their connection (n = 5; Fig 6G), and (8) middorsal stripe complete, all stripes widely fused on the tip of snout (n = 5; Fig 6H).

The ground dorsal color varies from reddish-brown (brick red, color 36 by Köhler [30]) to dark reddish-brown (vandyke brown, color 282 by Köhler [30]). Individuals show a light blue (light sky blue, color 191 by Köhler [30]) spot in the arms and in the thigh, in two individuals it was very diffuse. Limb coloration metallic varying from light coppery (light buff, color 2 by Köhler [30]) to orange coppery (light orange yellow, color 7 by Köhler [30]); limbs of two individuals were greenish (light olive yellow, color 117 by Köhler [30]). Depending on the variation in the size and quantity of ventral spots, light blue can prevail on the venter or can be suppressed by reddish-brown ventral spots.

### Advertisement call

The advertisement call of *Ranitomeya aetherea* **sp. nov.** (n = 23 calls of 7 males) consist of a cricket-like long-lasting trill of 26.1 ± 4.6 notes (16–35 notes; n = 23 calls, Fig 7) – most commonly of 21–29 notes (n = 16 calls). Calls have an average duration of 760 ± 120 ms (490–1,005 ms; n = 23 calls), and silence between calls of 14.2 ± 8.5 s (4.65–33.55 s; n = 16 intervals). Notes are tonal, have duration of 11.3 ± 1.38 ms (8.2–16.9 ms, n = 492 notes), silence between notes of 18.8 ± 2.91 ms (11.6–26.6 ms, n = 473 intervals), and a note rate of 33.6 ± 2.2 notes/s (30–36, n = 23 calls). Calls are emitted with a minimum frequency of 5,437 ± 171 Hz (4,603–5,751 Hz, n = 23 calls), a maximum frequency of 6,151 ± 135 Hz (5,415–6,441 Hz) and a dominant frequency of 5,815 ± 126 Hz (5,168–6,029 Hz, n = 23 calls; Fig 7). Frequently (60.9% of calls), the first 1–3 notes may be shorter (8–9 ms) than the others and have a lower dominant frequency that increases 100–200 Hz per note until it reaches the average values of the other notes. Acoustic characteristics of the advertisement call are summarized in Table 3.

**Table 3:** Acoustic characteristics of 23 analyzed advertisement calls of seven males of *Ranitomeya aetherea* sp. nov.

Variables	Mean	SD	Minimum	Maximum
CD – Call duration (ms)	760	120	490	1,005
SBC – Silence between calls (s)	14.2	8.47	4.65	33.55
NN – Number of notes per call	26.1	4.59	16	35
ND – Note duration (ms)	11.3	1.38	8.2	16.9
SBN – Silence between notes (ms)	18.8	2.91	11.6	26.6
NR – Note rate (notes per second)	33.6	2.15	30	36
LF – Minimum frequency (Hz)	5,437	171	4,603	5,751
HF – Maximum frequency (Hz)	6,151	135	5,415	6,441
DF – Dominant frequency (Hz)	5,815	127	5,168	6,029

SD = Standard Deviation

### Courtship call

We recorded a single male of *Ranitomeya aetherea* **sp. nov.** (MPEG 45225) emitting a series of 10 calls with distinct temporal and spectral parameters in the presence of a female, thus we considered it to be a courtship call. It consists of a long-lasting trill of 34.3 ± 4.03 notes (28–41 notes; n = 10 calls; Fig 8) most commonly of 31–39 notes (n = 8 calls) — a call duration of 1,130 ± 130 ms (919–1,345 ms; n = 10 calls) — and silence between calls of 11.0 ± 5.08 s (3.77–17.64 s; n = 9 intervals). Notes are tonal, have duration of 7.9 ± 1.94 ms (4.5–11.4 ms; n = 106 notes), a silence between notes of 25.7 ± 7.59 ms (12.9–53.1 ms; n = 96), and a note rate of 30.4 ± 1.6 notes/s (29–33 notes/s; n = 10 calls). Calls are emitted with a minimum frequency (LF) of 3,738 ± 686 Hz (3,041–5,033 Hz; n = 10 calls), a maximum frequency (HF) of 6,241 ± 334 Hz (5,796–7,102 Hz; n = 10 calls) and a dominant frequency (DF) of 5,469 ± 58 Hz (5,340–5,513 Hz; n = 10 calls). The notes can be divided into four groups, that doesn’t have a constant number of notes, but are easily distinguishable in the spectrogram: (1) 1–4 starting notes with increasing frequencies and shorter silence between notes; (2) notes with diffused spectral definition and higher silence between notes; (3) notes with diffused spectral definition and shorter silence between notes; and (4) notes with constant frequencies and shorter silence between notes, similar to advertisement call note. (Fig 8). Temporal and spectral traits are presented in Table 4.

**Table 4:** Acoustic variables of the courtship call of 10 analyzed calls of one male of *Ranitomeya aetherea* sp. nov. (MPEG 45225).

Variables	Mean	SD	Minimum	Maximum
CD – Call duration (ms)	1,130	130	919	1,345
SBC – Silence between calls (s)	11.02	5.08	3.77	17.64
NN – Number of notes per call	34.3	4.03	28	41
ND – Note duration (ms)	7.94	1.94	4.5	11.4
SBN – Silence between notes (ms)	25.68	7.59	12.9	53.1
NR – Note rate (notes per second)	30.4	1.59	29	34
LF – Minimum frequency (Hz)	3,738	686	3,041	5,533
HF – Maximum frequency (Hz)	6,240	334	5,797	7,102
DF – Dominant frequency (Hz)	5,469	58	5,340	5,512

Abbreviation: SD - Standard Deviation

### Tapdole

The description of tadpoles is based on five specimens (voucher INPA-H 47585) at Gosner [35] stages 26, 31, 37 (two tadpoles), and 38 (see Table 5). In stage 26 body ovoid in dorsal view and elliptical in lateral view, head wider than the rest of the body, snout rounded (Fig 9A). In stages 31, 37, and 38 body elliptical in dorsal and lateral view, head slightly wider than the rest of the body, snout rounded (Fig 9B). In all stages body dorsoventrally depressed. Body length 39.0–39.9% of the TL. Tail length is nearly 60% of TL. Eyes located dorsally, and directed dorsolaterally, ED 3.0–5.2% of the BL.

**Table 5:** Morphometric measurements (mm) of five tadpoles of *Ranitomeya aetherea* sp. nov.

Measurements (mm)	Tadpole stages				
	26	31	37	37	38
TL – Total length	14.5	16.9	22.1	23.0	23.2
BL – Body length	5.7	6.6	8.8	9.1	9.2
TAL – Tail length	8.8	10.3	13.3	13.9	14.0
BH – Body height	2.4	2.6	4.0	4.5	4.6
BW – Body width	3.2	3.7	4.9	5.5	6.3
BHN – Body heigth on the nostril	1.3	1.6	1.7	1.8	2.0
BHE – Body heigth on the eyes	1.8	2.4	3.0	2.9	3.1
BWN – Body width on the nostril	2.5	2.8	3.4	3.3	3.5
BWE – Body width on the eyes	3.1	3.6	4.6	4.5	4.6
TMW – Tail muscle width at base	1.0	1.3	1.8	1.8	1.9
MTH – Maximum tail height	1.9	2.4	3.5	3.5	3.5
DF – Dorsal fin height	0.5	0.6	0.9	0.9	0.9
VF – Ventral fin height	0.4	0.5	0.7	0.7	0.8
TMH – Tail muscle height	1.2	1.6	2.1	2.3	2.1
IOD – Interorbital distance	1.0	1.1	1.3	1.2	1.3
IND – Internarial distance	0.8	0.8	1.1	1.1	1.1
RED – Rostro-eye distance	1.6	1.7	2.1	2.2	2.3
RND – Rostro-nostril distance	0.9	1.0	1.2	1.4	1.4
RSD – Rostro-spiracle distance	3.5	3.8	4.1	4.2	4.2
ED – Eye diameter	0.4	0.7	1.0	1.1	1.2
END – Eye-nostril distance	1.0	1.1	1.3	1.2	1.3
SL – Spiracle length	0.5	0.7	0.9	1.1	1.0
SW – Spiracle width	0.3	0.4	0.6	0.7	0.7
SH – Spiracle height	0.5	0.7	1.2	1.5	1.6
VL – Vent tube length	0.4	0.5	0.6	0.7	0.7
ODW – Oral disc width	1.3	1.6	2.2	2.4	2.4
AL – Anterior (upper) labium	0.2	0.3	0.3	0.3	0.3
PL – Posterior (lower) labium	0.3	0.3	0.4	0.3	0.3
A1 – First anterior tooth row	1.2	1.7	–	2.3	2.7
A2 – Second anterior tooth row	0.9	0.6	–	0.8	0.8
A2 GAP – Medial gap in second anterior tooth row	0.4	0.6	–	0.8	1.2
P1 – First posterior tooth tor	0.9	1.5	–	2.0	2.0
P2 – Second posterior tooth row	1.0	1.3	–	2.0	2.0
P3 – Third posterior tooth row	–	1.3	–	1.7	1.7
P1 GAP – Medial gap in the first posterior tooth row	0.2	0.2	–	0.1	0.2
UJW – Upper jaw sheath width	0.8	1.0	1.1	1.0	1.1
UJL – Upper jaw sheath length	0.6	0.9	0.9	0.8	0.9

Nostril small elliptical, with slightly elevated marginal rim, located dorsally, directed anterolaterally, closer to the snout than to the eye in the early stages, gradually going closer to eyes in later stages. Spiracle sinistral, directed and opened posterodorsally, below the middle line of the body, SL 22.1–25.1% of BH. Spiracle visible ventrally and barely dorsally. Digestive tract visible in all stages, dark, folded, coiled ventrally, short, occupies less than half of the belly.

Caudal musculature robust, gradually tapering in the last quarter of tail, not reaching the tail tip; tail tip round (Fig 9A). Tail muscle width at base of tail 50–54% of the MTH. Dorsal fin begins near end of the body in stage 26 and posterior to the end of the body in other stages, very slightly arched, slightly higher than ventral fin, 38.6–43.6% of TMH. Ventral fin 32.0–35.8% of TMH.

Oral apparatus elliptical, located anteroventrally and visible laterally, slightly emarginate. The ratio of ODW/BW is 42% at stage 26. Anterior labium with groups of four (stage 26), or eight to nine (other stages) short, elliptical papillae, distributed in a single row on each side of the lateral margins, and split by a medial gap corresponding to approximately 3/4 of ODW. Posterior labium with a single row of 26–28 marginal short, elliptical, transparent papillae. Jaw sheath “U” shaped.

Lower jaw sheath narrower than upper jaw sheath, both serrated except for the extended tips of the upper jaw (Fig 9C). Upper jaw sheath, 43.2–59% of ODW. Labial tooth row formula 2(2)/2(1) in stage 26, and 2(2)/3(1) in the other stages; tooth row A1 three to five teeth longer than A2. A2 with a medial gap, size of the medial gap is equal to the size of each side of A2 tooth row. P1 and P2 are similar in length, P3 always shorter than P1 and P2 when present.

**Coloration.** After four months of preservation in 10% formalin, the tadpoles have whitish to cream (cream, color 12 by Köhler [30]) ground coloration with fine brown (antique brown, color 300 by Köhler [30]) reticulations. Venter is translucent, the gut is black. Iris is black. Spiracle translucent (Fig 9).

In life, the entire body and tail have a transparent layer containing a light reddish-brown (orange rufous, color 56 by Köhler [30]) reticulation. Head translucent grey, body gray (pratt’s payne gray, color 293 by Köhler [30]), eyes black. Abdomen mostly transparent, digestive tract and heart visible. Tail musculature light red brown (salmon color, color 58 by Köhler [30]), dorsal and ventral fins transparent with fine light red brown (orange rufous, color 56 by Köhler [30]) reticulation (denser in dorsal fin). In older stages (37 and 38) middorsal light blue (light-sky blue, color 191 by Köhler [30]) stripe begins to appear. Metamorphosed individuals already bear the color pattern of adult frogs.

### Diagnosis

The new species is compared to all other currently recognized *Ranitomeya* species: *R. amazonica* (Schulte 1999 [17]), *R. benedicta* Brown, Twomey, Pepper & Sanchez-Rodriguez 2008 [59], *R. cyanovittata* Peres-Peña, Chávez, Twomey & Brown 2010 [12], *R. defleri* Twomney & Brown 2009 [60], *R. fantastica* (Boulenger 1884 [61]), *R. flavovittata* (Schulte 1999 [17]), *R. imitator* (Schulte 1986 [62]), *R. reticulata* (Boulenger 1884 [61]), *R. sirensis* (Aichinger 1991 [63]), *R. summersi* Brown, Twomey, Pepper & Sanchez-Rodriguez 2008 [59], *R. toraro* Brown, Caldwell, Twomey, Melo-Sampaio & Souza 2011 [4], *R. uakarii* Brown, Schulte & Summers 2006 [64], *R. vanzolinii* (Myers 1982 [65]), *R. variabilis* (Zimmermann & Zimmermann 1988 [66]), *R. ventrimaculata* (Shreve 1935 [67]), and *R. yavaricola* Peres-Peña, Chávez, Twomey & Brown 2010 [12]. Additionally, we compared the new species to *Ranitomeya* sp., currently under description [58]. We gave more details on the comparison with the closest relatives (with lower genetic distance), *R. cyanovittata* and *R. yavaricola*.

*Ranitomeya aetherea* **sp. nov.** is readily distinguished by its unique color pattern (light sky-blue stripes on dark reddish-brown dorsum and light reddish-brown spotting on metallic pale orange yellow limbs) from all species that have yellow, orange and red dorsal patterns, or reticulated limb patterns by its unique color pattern of light sky-blue stripes on dark reddish-brown dorsum and light reddish-brown spotting on metallic pale orange yellow limbs. The new species is similar to *Ranitomeya* sp. but differs from it by its light sky-blue dorsal stripes (light yellowish green to light metallic turquoise-green in *Ranitomeya* sp.) and by the absence of a conspicuous sulfur yellow ocellus-like spot on the dorsal surface of the thighs (present on *Ranitomeya* sp.). The two most similar species (*R. cyanovittata* and *R. yavaricola*) have pale turquoise blue dorsal stripes or spots but *R. aetherea* **sp. nov.** differs from both by its metallic pale orange yellow limbs with light reddish-brown spots (limbs with turquoise blue stripes and bars on black in *R. cyanovittata* [12], and patternless bronze in *R. yavaricola* [12]). In addition, *R. aetherea* **sp. nov.** has poorly developed lateral fringes on the toes (absent in other species).

Has smaller male SVL (15.2–16.9 mm) than *R. fantastica* (approx. 20 mm [61]), *R. imitator* (approx. 19 mm [62]), *R. summersi* (17.5–19.5 mm [59]), *R. vanzolinii* (16.7–18.8 mm [65]), larger male SVL than *R. cyanovittata* (13.8 mm [12]), *R. sirensis* (14.7–15.4 mm [63]), *R. toraro* (14.8–15.6 mm [4]), and *R. uakari* (14.8–15.5 mm [64]), and smaller female SVL (14.4–16.9 mm) than *R. benedicta* (16.8–20.2 mm [59]), *R. cyanovittata* (17.3 mm [12]), *Ranitomeya* sp. (17.3–18.5 mm [58] and *R. vanzolinii* (16.8–19 mm [65]).

The new species distinguishes also by its greater male head width (5.0–5.7 mm) from *R. cyanovittata* (3.9 mm [12]), *R. sirensis* (4.7–5.0 mm [63]), *R. toraro* (4.7–5.1 mm [4]), and by smaller head width from *R. imitator* (approx. 6.0 mm [62]); it differs by greater male head length (4.1–4.8 mm) from *R. cyanovittata* (3.6 mm [12]) and *R. sirensis* (3.0–3.8 mm [63]), and by smaller male head length from *R. benedicta* (5.1–5.9 mm [59]), *R. defleri* (5.0–7.2 mm [60]), *R. imitator* (approx. 6 mm [62]), *Ranitomeya* sp. (4.8–5.4 mm [58]), *R. summersi* (5.1–5.3 mm [59]), *R. uakarii* (4.9–5.4 [64]), *R. ventrimaculata* (5.0–6.0 mm [67]) and *R. yavaricola* (5.5–6.6 mm [12]); finally it differs by its smaller female head length (3.9–4.5 mm) from *R. cyanovittata* (5.1 mm [12]), *Ranitomeya* sp. (5.1–5.2 mm [58]), *R. toraro* (5.5 mm [4]), *R. uakarii* (4.9–5.5 mm [64]) and *R. yavaricola* (5.9–6.3 mm [12]).

Additional differences can be found in the skin texture of the ventral parts of the body. The belly skin is shagreen to weakly granular of *Ranitomeya aetherea* **sp. nov.** in the center, but granular between the arms, near to the cloaca and to the flanks (granular skin forms ring shaped region). The belly skin texture of the other species is as follows: broadly smooth in *R. uakari* [64], weakly granular in *R. benedicta* [59], broadly weakly granular in *R. cyanovittata* [12], *R. defleri* [60], *R. sirensis R. sumersi* [59], *R. toraro* [4], and *R. yavaricola* [12], shagreen in *Ranitomeya* sp. [58], broadly moderately granular in *R. sirensis* [63], and broadly granular in *R. vanzolinii* [65].

Comparing with available literature data, the advertisement call of *R. aetherea* **sp. nov.** cannot be distinguished from the advertisement calls described for *R. imitator* and *R. yavaricola* [4,12]. Nevertheless, the information about note duration, silence between notes and minimum and maximum frequencies is missing in these species. Moreover, advertisement calls of *R. cyanovittata* and *R. toraro* remain unknown. In comparison with other *Ranitomeya* species, the advertisement call of *R. aetherea* **sp. nov.** shows high similarity to the advertisement calls of *R. flavovittata*, *R. sirensis*, and *R. vanzolinii*, which are also characterized by long-lasting trills. However, the call of *R. aetherea* **sp. nov.** differs in higher note rate 30–36 notes/s (29–30 notes/s in *R. flavovittata* [4]; 24–30 notes/s in *R. sirensis* [4]; and 26–28 notes/s in *R. vanzolinii* [4].

From the remaining *Ranitomeya* species *R. aetherea* **sp. nov.** differs as follows: from *R. amazonica* by longer duration of the calls (0.49–1.01 vs. 0.16–0.36 s) and lower note rate (30–36 notes/s vs. 85–138 notes/s [4]), from *R. benedicta* by longer duration of the calls (0.49–1.01 s vs. 0.10–0.17 s) and higher dominant frequency (5513–6029 Hz vs. 3190–4240 Hz [59]), from *R. defleri* by lower number of notes (16–35 vs. 40–61), lower note rate (30–36 notes/s vs. 94–104 notes/s), and higher dominant frequency (5513–6029 Hz vs. 5319–5414 Hz [60]), from *R. fantastica* by longer duration of the calls (0.49–1.01 s vs. 0.18–0.32 s [4]), higher number of notes (16–35 vs. 10–13), lower note rate (30–36 notes/s vs. 41–57 notes/s), and higher dominant frequency (5513–6029 Hz vs. 2950–3790 Hz [4]), from *R. reticulata* by longer duration of the calls (0.49–1.01 s vs.0.18–0.29 s), lower number of notes (16–35 vs. 48–94), lower note rate (30–36 notes/s vs. 270–382 notes/s), and higher dominant frequency (5513–6029 Hz vs. 4140–4480 Hz [4]), from *R. summersi* by longer duration of the calls (0.49–1.01 s vs. 0.38–0.50 s), higher number of notes (16–35 vs. 14–16), lower note rate (30–36 notes/s vs. 39–40 notes/s), and higher dominant frequency (5513–6029 Hz vs. 2760–3220 Hz [59]), from *R. uakarii* by longer duration of the calls (0.49–1.01 s vs. 0.26–0.29 s), higher number of notes (16–35 vs. 14–16), lower note rate (30–36 notes/s vs. 50–58 notes/s), and higher dominant frequency (5513–6029 Hz vs. 3790–4130 Hz [4,64]), from *R. variabilis* by longer duration of the calls (0.49–1.01 s vs. 0.14–0.44 s), lower note rate (30–36 notes/s vs. 106–297 notes/s), and higher dominant frequency (5513–6029 Hz vs. 4386–5624 Hz [4]), from *R. ventrimaculata* by longer duration of the calls (0.49–1.01 s vs. 0.32–0.38 s), lower number of notes (16–35 vs.58–63), lower note rate (30–36 notes/s vs. 166–181 notes/s), and higher dominant frequency (5513–6029 Hz vs. 4190–4400 Hz [4]).

We found information on the tadpoles for eleven *Ranitomeya* species. Among the known tadpoles, some descriptions are based on one tadpole or on back-riding tadpoles, thus we compare the species using the ratios between measurements (values of compared species in parentheses). The ratio of the tail length/total length is 60% in all stages of *R*. *aetherea* **sp. nov.**: it is larger than *R. amazonica* (45% st. 29 [4]), *R. flavovittata* (57% st. 26 [4]), *R. reticulata* (41% st. 30 [4]) and *R. variabilis* (52% st. 28 [4]); it is smaller than *R. defleri* (64% st. 30 [4]), *R. toraro* (64.2% st. 25 [4]), *Ranitomeya* sp. (63–64% in all stages [58]), *R. vanzolinii* (67.9% st. 38 [4]), *R. imitator* (62% st. 26 [4]), *R. uakarii* (62%, st. 29 [4]) and *R. yavaricola* (62% st. 25 [12]). The labial tooth row formula in *R. aetherea* **sp. nov.** 2(2)/2(1) st. 26 differs from formula 2(2)/3(1) known for *Ranitomeya* sp. all stages [58] and *R. yavaricola* st. 25 [12]. Posterior tooth rows of *R. aetherea* sp. nov. P1 < P2 (P1 = 87% of P2), st. 26 and P1 = P2 > P3 (P3 = 83% to 87% of P1), st. 31, 37 and 38 differ from rows known for *R. amazonica* (P1 = P2 > P3, P3 = 80% of P1, st. 26 [4]), *R. flavovittata* (P1 = P2 > P3, P3 = 80% of P1, st. 26 [4]), *R. imitator* (P1 = P2 > P3, P3 = 55% of P1, st. 26 [4,68]), *R. reticulata* (P1 = P2 > P3, P3 = 80% of P1, st. 30 [4]), *Ranitomeya* sp. (P1 > P2 > P3, P3 = 78–88% of P1), *R. toraro* (P1 > P2, st.25 [4]), *R. uakarii* (P1 = P2 > P3, P3 = 75% of P1, st. 29 [4]), *R. vanzolinii* (P1 < P2 = P3, P1 = 44.6% of P2, st. 38 [4]), and *R. variabilis* (P1 = P2 > P3, P3 = 75% of P2; st. 30 [4]). In life, the tadpoles of *R. aetherea* **sp. nov.** in the final stages differ from described tadpoles of all other species by having light bluish gray dorsal stripes.

### Distribution, natural history and conservation

*Ranitomeya aetherea* **sp. nov.** is currently known only from the type locality, on preserved forests next to the Juruá River, in the Comunidade de Nova Esperança, municipality of Eirunepé, state of Amazonas, Brazil (Fig 10). We have sampled four RAPELD modules in the region, and the new species has only been recorded at just one site – module 2.

We have found the new species living in sympatry with *Ranitomeya* cf. *toraro* and other Dendrobatoidea: *Allobates femoralis*, *Allobates* aff. *velocicantus*, *Allobates* sp. undescribed species (A.P. Lima, unpublished data), *Ameerega hahneli* and *Ameerega trivittata*.

*Ranitomeya aetherea* **sp. nov.** is a diurnal species, active mostly in the early morning and late afternoon. When it rains, it may be active all day. *Ranitomeya aetherea* **sp. nov.** was encountered in an area of open ombrophylous forest with palms (Fig 11A). It was usually associated with ‘*banananeira brava*’ plants (*Phenakospermum guyannense*, Strelitziacaea; Fig 11B) or fallen leaves of palm trees. In the early morning before the beginning of call activity the individual frogs were spotted up to 4 m above the ground. When the calling activity began, they usually agilely climbed down the *P. guyannense* pseudostem. Foraging individuals were observed both on the *P. guyannense* pseudostem and in the ground litter.

Although we did not find egg clutches in the nature, a female had deposited a single unfertilized egg on the wall of the transport container. The egg was small and brown, covered in a thick transparent gelatinous layer.

The tadpoles were deposited in various phytotelmata, most commonly in the water accumulated in the *P. guyannense* axils or in small tree holes (Fig 11C). Only one tadpole appears to be deposited to each phytotelma, as we have never found more tadpoles together. On the other hand, more tadpoles may develop in a single plant if it provides a higher number of phytotelmata. Older tadpoles start to display the adult color pattern (Fig 11D) and the color development is almost complete during metamorphosis (Fig 11E). The finding of tadpoles of different stages together with metamorphosed individuals in the same area indicates that reproduction of *R. aetherea* **sp. nov.** has a prolonged character. In some cases, tadpoles of *R.* cf. *toraro* developed in the same plant as tadpoles of the new species.

Calling males were perched on various plant leaves, dry leaves, or in leaf sheaths of *Phenakospermum* 60–300 cm above the ground. (Fig 11F and 11G). Calling activity started during the dawn (ca. 6 a.m.) and lasted until ca. 10 a.m., having a peak between 6:30–8:00 a.m. A second period of less intense calling activity was observed between ca.16–18:30 p.m., ending with dusk. Adult males were distributed evenly in the whole study area. However, sometimes multiple males can be found on a single *P. guyannense* plant. Individual females were usually present near these male groups. When the females approached a male, the male started its courtship call. This behavior suggests that the species has a promiscuous mating system. Males appear to be territorial, and they respond and approach the playback of their advertisement call.

According to our observations, individuals of *Ranitomeya* cf. *toraro* occupy the same plants as *R. aetherea* **sp. nov.** and may use the same shelters and breeding sites.

## Discussion

We recovered *Ranitomeya* genus as monophyletic with a posterior probability of 1 and mostly congruent with the revisions of Grant et al. [69], Brown et al. [4] and with some distinctions to the genomic framework of Muell et al. [11]. We recovered the same groups as defined in Brown et al. [4]: *R. defleri*, *R. variabilis*, *R. reticulata* and *R. vanzolinii*. But a low posterior probability between *R. defleri* and *R. toraro* points out to the findings of Muell et al. [11] that the *R. defleri* group could be non-monophyletic.

The species of the *Ranitomeya vanzolinii* species group are distributed across the Southwestern Amazonia [10,11] but most of them have a narrow distribution range. *Ranitomeya aetherea* **sp. nov.** is currently known only from its type locality lying at the left bank of the Juruá River. Another possible locality of the occurrence of *R. aetherea* **sp. nov.** (species tentatively determined as *R.* cf. *yavaricola*) was reported from the surroundings of Eirunepé City (i.e., 50 km NE of the type locality [57]). Therefore, it seems that also *R. aetherea* **sp. nov.** is restricted to small distribution range. However, the Juruá River area is very poorly studied [18,19] and it will not be a surprise if future research provides evidence of a wider occurrence of the new species.

The 16S genetic distances found between individual *Ranitomeya* species are not as high as the distances usually found in other Neotropical anuran species (e.g., Fouquet et al. [70] Vacher et al. [71]). Between some *Ranitomeya* species the values of 16S p-distances are around 2%. Nevertheless, except for *R. sirensis*, all species also show low intraspecific p-distances showing a significative structuration even though the low interspecific distances. According to our knowledge, most *Ranitomeya* species have a small geographic range. Therefore, also the available genetic data covers very small areas. This fact may raise the question whether the differences between the samples from an isolation-by-distance system do not represent a cline variation. But as stated in Brown et al. [4] the size of the distribution ranges of species of the genus *Ranitomeya* appears to have little relation to species delimitation. However, further sampling specially in Brazilian western Amazonia would help to improve knowledge of the distribution and relationships of species in the genus *Ranitomeya*.

According to our data the new species is closely related to *R. cyanovittata* (16S p-distance 2.04). However, *R. aetherea* **sp. nov.** was already included in a phylogenetic framework based on genomic data (under the tentative name *R.* cf. *yavaricola*, see Twomey et al. [57]), where it showed lower relatedness to *R. cyanovittata* than in our mitochondrial framework [57]. This finding supports the species status of *R. aetherea* **sp. nov.** despite its lower mitochondrial distance from *R. cyanovittata*.

Even after more than a decade since the last species descriptions [4], the genus *Ranitomeya* has been largely studied for its color patterns [57,70–72]. It has been noted that color morphs do not always correspond to species delimitation due to many instances of mimicry and intraspecific variability [15,73–75]. Although the recent descriptions [4,12,58] and especially revisions made by Brown et al. [4] and Muell et al. [11] present valuable data on the morphology, call, tadpoles, and inter- and intraspecific relationships, all the descriptions up to date are based mostly on the comparisons of the species color patterns. There are also some biases with the data of many species. Description of one species (*R. flavovittata*) is based on a single juvenile, nine of the 16 species have less than seven type specimens, and in nearly half of the species almost no morphometric data are available in their original descriptions. Thus, despite the progress already achieved by Brown et al. [4] and Muell et al. [11], there is a need to redefine most species of *Ranitomeya* using an integrative approach combining precise morphological and modern bioacoustic and genetic methods. Such a step would greatly facilitate the work of taxonomists on the descriptions of other new species of the genus *Ranitomeya*.

## Supporting information

S1 Table Morphometric measurements (in mm) of adults of the type series of *Ranitomeya aetherea* sp. nov. Measurement acronyms are defined in the text. Abbreviations: INPA-H, Instituto Nacional de Pesquisas da Amazônia; MPEG, Museu Paraense Emílio Goeldi; FN, field numbers; M, male; F, female.
(DOCX)

S2 Table Acoustic parameters of call of *Ranitomeya aetherea* sp. nov. Advertisement and courtship (bold) calls. Abbreviations: vouchers: INPA-H, Instituto Nacional de Pesquisas da Amazônia; MPEG, Museu Paraense Emílio Goeldi; FNJV, Fonoteca Neotropical Jacques Vielliard; AT, air temperature (ºC); NN, number of notes per call; CD, call duration (ms); SBC, silence between calls (s); ND, note duration (ms); SBN, silence between notes (ms); LF, minimum frequency (Hz); HF, maximum frequency (Hz); and DF, dominant frequency (Hz).
(DOCX)

S3 Table Specimens of *Ranitomeya*, *Andinobates* and *Excidobates* used in phylogenetic analyses. (DOCX)

S4 Table Species delimitation results of Ranitomeya. (XLSX)

S5 Table Interspecific and intraspecific genetic distances (12S) between *Ranitomeya aetherea* sp. nov. and closely related taxa. (XLSX)

S6 Table Interspecific and intraspecific genetic distances (CYTB) between *Ranitomeya aetherea* sp. nov. and closely related taxa. (XLSX)

S1 Fig Schematic drawings of the measurement taken from the new species. (TIF)

S2 Fig Phylogenetic reconstruction showing the position of *Ranitomeya aetherea* sp. nov. (See Fig 1). Bayesian inference tree inferred with 16S, 12S, COI and CytB. Non-parametric bootstrap support is shown close to nodes.
(TIF)
