# Supplementary material for: A remarkable new blue Ranitomeya species (Anura: Dendrobatidae) with copper metallic legs from open forests of Juruá River Basin, Amazonia
Source: PLoS One. 2025 May 14;20(5):e0321748. doi: 10.1371/journal.pone.0321748 (PMC12077741; doi:10.1371/journal.pone.0321748)
Supplement: S1 Table — Measurement acronyms are defined in the text. Abbreviations: INPA-H, Instituto Nacional de Pesquisas da Amazônia; MPEG, Museu Paraense Emílio Goeldi; FN, field numbers; M, male; F, female. (DOCX) [file pone.0321748.s001.docx]

**S1 Table. Morphometric measurements (in mm) of adults of the type series of *Ranitomeya aetherea* sp. nov.**

Measurement acronyms are defined in the text. Abbreviations: INPA-H, Instituto Nacional de Pesquisas da Amazônia; MPEG, Museu Paraense Emílio Goeldi; FN, field numbers; M, male; F, female.

*(1/2)*

| **Voucher** | **Sex** | **SVL** | **HL** | **HW** | **IOD** | **UEW** | **MTD** | **TD** | **DET** | **ED** | **SL** | **END** | **BW** | **TSCN** | **IND** | **FL** | **TL** | **TaL** | **KK** | **FoL** | **LT1** | **LT2** | **LT3** | **LT5** |
| --- | --- | --- | --- | --- | --- | --- | --- | --- | --- | --- | --- | --- | --- | --- | --- | --- | --- | --- | --- | --- | --- | --- | --- | --- |
| MPEG 45224 | F | 15.85 | 4.27 | 5.29 | 1.97 | 1.41 | 0.67 | 0.97 | 0.47 | 2.16 | 1.83 | 1.30 | 4.92 | 0.96 | 1.87 | 6.84 | 6.39 | 4.08 | 13.72 | 6.03 | 1.85 | 3.06 | 4.80 | 4.04 |
| MPEG 45225 | M | 15.81 | 4.05 | 5.18 | 1.80 | 1.23 | 0.53 | 0.92 | 0.46 | 2.01 | 1.85 | 1.35 | 5.04 | 1.02 | 1.89 | 6.78 | 6.71 | 4.00 | 13.81 | 6.17 | 1.77 | 2.76 | 4.56 | 4.14 |
| MPEG 45226 | M | 15.62 | 4.14 | 5.18 | 1.77 | 1.17 | 0.47 | 0.72 | 0.57 | 2.18 | 1.77 | 1.30 | 4.64 | 0.92 | 1.84 | 6.42 | 6.39 | 4.07 | 13.20 | 5.80 | 1.20 | 2.49 | 4.09 | 3.67 |
| MPEG 45227 | M | 15.91 | 4.19 | 5.36 | 2.00 | 1.24 | 0.61 | 0.86 | 0.57 | 2.06 | 1.89 | 1.34 | 5.11 | 1.03 | 1.90 | 6.74 | 6.31 | 4.10 | 13.40 | 5.56 | 1.49 | 2.72 | 4.20 | 3.78 |
| INPA-H 47571 | M | 16.13 | 4.17 | 5.43 | 1.98 | 1.24 | 0.63 | 0.74 | 0.58 | 1.97 | 1.90 | 1.49 | 4.99 | 1.01 | 2.01 | 6.80 | 6.43 | 4.32 | 13.72 | 5.97 | 1.68 | 2.87 | 4.54 | 4.02 |
| INPA-H 47572 | M | 16.15 | 4.41 | 5.35 | 2.05 | 1.31 | 0.41 | 0.91 | 0.63 | 2.01 | 1.78 | 1.31 | 5.11 | 1.07 | 2.03 | 6.79 | 6.47 | 4.06 | 13.52 | 5.77 | 1.64 | 2.70 | 4.25 | 3.77 |
| INPA-H 47573 | M | 15.64 | 4.11 | 5.00 | 1.95 | 1.08 | 0.51 | 0.72 | 0.59 | 1.89 | 1.80 | 1.27 | 5.13 | 1.07 | 1.97 | 6.50 | 6.12 | 3.88 | 13.26 | 5.63 | 1.69 | 2.82 | 4.44 | 3.81 |
| INPA-H 47574 | M | 15.85 | 4.21 | 5.37 | 2.04 | 1.05 | 0.63 | 0.73 | 0.61 | 1.97 | 1.76 | 1.32 | 5.12 | 0.95 | 1.93 | 6.55 | 6.33 | 4.08 | 13.37 | 5.33 | 1.50 | 2.54 | 4.13 | 3.55 |
| INPA-H 47575 | M | 15.90 | 4.17 | 5.51 | 2.06 | 1.36 | 0.54 | 0.89 | 0.51 | 2.05 | 1.72 | 1.27 | 5.11 | 0.97 | 1.94 | 6.41 | 6.37 | 4.32 | 13.00 | 5.85 | 1.54 | 2.80 | 4.46 | 4.03 |
| INPA-H 47576 | M | 15.77 | 4.45 | 5.23 | 1.96 | 1.34 | 0.56 | 0.91 | 0.50 | 1.99 | 1.89 | 1.34 | 5.06 | 1.09 | 1.95 | 6.79 | 6.51 | 3.96 | 13.41 | 5.89 | 1.44 | 2.73 | 4.42 | 3.98 |
| INPA-H 47577 | M | 16.42 | 4.74 | 5.33 | 2.00 | 1.36 | 0.60 | 0.91 | 0.57 | 1.97 | 1.74 | 1.35 | 5.37 | 1.04 | 2.05 | 6.88 | 6.55 | 4.39 | 14.00 | 6.01 | 1.59 | 2.86 | 4.53 | 4.08 |
| INPA-H 47578 | M | 15.38 | 4.08 | 5.45 | 1.61 | 1.30 | 0.59 | 0.67 | 0.47 | 1.89 | 1.87 | 1.42 | 5.02 | 1.00 | 1.92 | 6.23 | 5.69 | 3.29 | 12.33 | 5.42 | 1.48 | 2.66 | 4.10 | 3.70 |
| INPA-H 47579 | M | 16.99 | 4.79 | 5.68 | 2.16 | 1.43 | 0.51 | 1.08 | 0.59 | 2.04 | 1.92 | 1.48 | 5.70 | 1.18 | 2.17 | 7.15 | 6.68 | 4.41 | 14.35 | 6.53 | 1.61 | 3.06 | 4.88 | 4.42 |
| INPA-H 47580 | F | 16.47 | 4.33 | 5.29 | 1.87 | 1.31 | 0.58 | 1.03 | 0.48 | 2.02 | 1.97 | 1.44 | 5.59 | 0.98 | 1.90 | 6.65 | 6.10 | 4.05 | 13.23 | 5.91 | 1.55 | 2.69 | 4.44 | 3.92 |
| INPA-H 47581 | M | 15.69 | 4.15 | 5.61 | 1.82 | 1.39 | 0.56 | 0.99 | 0.53 | 2.13 | 2.05 | 1.54 | 5.36 | 1.08 | 2.02 | 6.55 | 6.36 | 3.94 | 13.31 | 5.84 | 1.49 | 2.70 | 4.19 | 3.96 |
| INPA-H 47582 | M | 16.63 | 4.65 | 5.40 | 2.03 | 1.29 | 0.56 | 1.21 | 0.59 | 1.94 | 2.16 | 1.46 | 5.45 | 1.10 | 2.04 | 6.87 | 6.40 | 4.14 | 13.47 | 6.08 | 1.55 | 2.93 | 4.43 | 4.14 |
| INPA-H 47583 | F | 16.49 | 4.57 | 5.29 | 1.88 | 1.33 | 0.61 | 0.80 | 0.57 | 1.97 | 1.99 | 1.49 | 5.83 | 1.10 | 2.12 | 7.14 | 7.01 | 4.56 | 15.26 | 7.05 | 1.73 | 3.31 | 5.09 | 4.82 |
| INPA-H 47584 | F | 16.92 | 4.45 | 5.56 | 2.15 | 1.13 | 0.51 | 0.91 | 0.59 | 1.97 | 1.89 | 1.42 | 5.49 | 1.09 | 2.04 | 6.85 | 6.42 | 4.03 | 13.51 | 6.25 | 1.53 | 2.95 | 4.64 | 4.35 |
| INPA-H 47586 | M | 16.19 | 4.12 | 5.19 | 2.00 | 1.22 | 0.58 | 0.69 | 0.61 | 2.15 | 1.75 | 1.25 | 5.42 | 1.07 | 2.02 | 6.91 | 6.47 | 4.07 | 14.06 | 6.23 | 1.51 | 2.77 | 4.39 | 4.31 |
| INPA-H 47587 | M | 15.22 | 4.36 | 5.37 | 1.82 | 1.25 | 0.69 | 0.90 | 0.51 | 2.01 | 1.86 | 1.35 | 5.10 | 0.98 | 1.93 | 6.77 | 6.55 | 4.09 | 13.42 | 5.68 | 1.47 | 2.59 | 4.17 | 3.90 |
| INPA-H 47588 | M | 15.39 | 4.15 | 5.21 | 2.04 | 1.09 | 0.59 | 0.77 | 0.58 | 1.91 | 1.55 | 1.23 | 5.40 | 0.91 | 1.84 | 6.56 | 6.15 | 3.83 | 13.16 | 5.72 | 1.55 | 2.70 | 4.25 | 4.00 |
| INPA-H 47589 | M | 15.62 | 4.19 | 5.32 | 1.95 | 1.27 | 0.74 | 0.83 | 0.46 | 2.09 | 1.61 | 1.28 | 5.29 | 1.07 | 2.04 | 6.73 | 6.25 | 4.03 | 13.40 | 6.16 | 1.54 | 2.74 | 4.43 | 4.23 |
| INPA-H 47590 | F | 16.33 | 4.48 | 5.27 | 1.86 | 1.30 | 0.47 | 0.84 | 0.54 | 2.16 | 1.73 | 1.33 | 6.07 | 1.07 | 2.06 | 6.93 | 6.40 | 4.05 | 13.75 | 6.09 | 1.51 | 2.74 | 4.52 | 4.02 |
| MPEG 45228 | F | 16.10 | 4.05 | 5.22 | 2.11 | 1.24 | 0.63 | 0.98 | 0.48 | 2.06 | 1.97 | 1.47 | 5.85 | 1.12 | 2.11 | 7.22 | 6.23 | 3.84 | 14.14 | 5.92 | 1.53 | 2.69 | 4.54 | 3.90 |
| MPEG 45229 | M | 15.35 | 4.18 | 5.19 | 1.78 | 1.35 | 0.61 | 0.86 | 0.50 | 1.94 | 1.75 | 1.34 | 5.03 | 0.93 | 1.87 | 6.91 | 6.13 | 3.73 | 13.91 | 5.80 | 1.30 | 2.42 | 4.14 | 3.89 |
| INPA-H 47591 | F | 14.35 | 3.92 | 4.91 | 1.59 | 1.17 | 0.72 | 0.73 | 0.64 | 1.81 | 1.37 | 0.97 | 4.93 | 1.05 | 1.87 | 6.12 | 5.42 | 3.49 | 12.36 | 5.38 | 1.17 | 2.29 | 3.98 | 3.65 |

*(2/2)*

| **Voucher** | **W1TD** | **W1T** | **W2TD** | **W2T** | **W3TD** | **W3T** | **W4TD** | **W4T** | **W5TD** | **W5T** | **AL** | **FAL** | **HaL** | **L1F** | **L2F** | **L4F** | **W1FD** | **W1F** | **W2FD** | **W2F** | **W3FD** | **W3F** | **W4FD** | **W4F** |
| --- | --- | --- | --- | --- | --- | --- | --- | --- | --- | --- | --- | --- | --- | --- | --- | --- | --- | --- | --- | --- | --- | --- | --- | --- |
| MPEG 45224 | 0.29 | 0.25 | 0.48 | 0.35 | 0.46 | 0.43 | 0.68 | 0.50 | 0.64 | 0.50 | 4.73 | 3.92 | 4.19 | 1.78 | 2.82 | 3.16 | 0.38 | 0.31 | 0.52 | 0.40 | 0.75 | 0.61 | 0.60 | 0.43 |
| MPEG 45225 | 0.35 | 0.31 | 0.46 | 0.36 | 0.54 | 0.42 | 0.73 | 0.55 | 0.70 | 0.60 | 4.57 | 3.51 | 4.16 | 1.66 | 2.94 | 3.47 | 0.45 | 0.32 | 0.66 | 0.49 | 0.78 | 0.58 | 0.81 | 0.65 |
| MPEG 45226 | 0.34 | 0.30 | 0.40 | 0.37 | 0.59 | 0.48 | 0.56 | 0.51 | 0.51 | 0.43 | 4.58 | 3.62 | 4.06 | 1.61 | 2.57 | 3.16 | 0.36 | 0.30 | 0.52 | 0.35 | 0.63 | 0.49 | 0.54 | 0.41 |
| MPEG 45227 | 0.36 | 0.38 | 0.49 | 0.44 | 0.67 | 0.55 | 0.80 | 0.58 | 0.76 | 0.60 | 4.26 | 3.40 | 3.93 | 1.68 | 2.65 | 3.06 | 0.35 | 0.33 | 0.66 | 0.44 | 0.92 | 0.62 | 0.83 | 0.58 |
| INPA-H 47571 | 0.36 | 0.33 | 0.48 | 0.45 | 0.63 | 0.54 | 0.73 | 0.62 | 0.67 | 0.61 | 4.72 | 3.66 | 4.12 | 1.80 | 2.82 | 3.28 | 0.45 | 0.37 | 0.63 | 0.47 | 0.82 | 0.56 | 0.75 | 0.48 |
| INPA-H 47572 | 0.30 | 0.25 | 0.43 | 0.38 | 0.56 | 0.49 | 0.76 | 0.60 | 0.65 | 0.59 | 4.53 | 3.82 | 4.03 | 1.66 | 2.62 | 3.18 | 0.45 | 0.40 | 0.67 | 0.46 | 0.98 | 0.67 | 0.87 | 0.70 |
| INPA-H 47573 | 0.30 | 0.27 | 0.53 | 0.44 | 0.63 | 0.48 | 0.76 | 0.53 | 0.79 | 0.66 | 4.38 | 3.65 | 4.29 | 1.64 | 2.83 | 3.11 | 0.44 | 0.34 | 0.63 | 0.48 | 0.90 | 0.67 | 0.81 | 0.61 |
| INPA-H 47574 | 0.29 | 0.27 | 0.45 | 0.40 | 0.60 | 0.48 | 0.62 | 0.50 | 0.71 | 0.59 | 4.62 | 3.67 | 3.96 | 1.64 | 2.36 | 2.89 | 0.32 | 0.30 | 0.51 | 0.45 | 0.70 | 0.57 | 0.77 | 0.57 |
| INPA-H 47575 | 0.36 | 0.35 | 0.56 | 0.46 | 0.73 | 0.58 | 0.81 | 0.62 | 0.79 | 0.60 | 4.62 | 3.84 | 4.20 | 1.77 | 2.94 | 3.34 | 0.41 | 0.37 | 0.69 | 0.58 | 0.88 | 0.69 | 0.82 | 0.55 |
| INPA-H 47576 | 0.40 | 0.40 | 0.51 | 0.44 | 0.69 | 0.53 | 0.88 | 0.73 | 0.69 | 0.56 | 4.49 | 3.75 | 4.08 | 1.64 | 2.90 | 3.20 | 0.46 | 0.43 | 0.67 | 0.53 | 0.97 | 0.65 | 0.89 | 0.61 |
| INPA-H 47577 | 0.41 | 0.39 | 0.53 | 0.42 | 0.76 | 0.59 | 1.05 | 0.77 | 0.90 | 0.63 | 4.78 | 3.83 | 4.26 | 1.84 | 2.81 | 3.26 | 0.45 | 0.37 | 0.71 | 0.47 | 1.06 | 0.71 | 0.99 | 0.67 |
| INPA-H 47578 | 0.39 | 0.39 | 0.50 | 0.45 | 0.69 | 0.58 | 0.82 | 0.67 | 0.78 | 0.65 | 4.08 | 3.76 | 3.84 | 1.63 | 2.66 | 3.02 | 0.40 | 0.35 | 0.66 | 0.54 | 0.95 | 0.69 | 0.85 | 0.63 |
| INPA-H 47579 | 0.41 | 0.35 | 0.53 | 0.46 | 0.65 | 0.60 | 0.87 | 0.74 | 0.81 | 0.72 | 4.72 | 3.99 | 4.52 | 1.96 | 3.04 | 3.44 | 0.41 | 0.39 | 0.65 | 0.60 | 0.96 | 0.82 | 0.81 | 0.70 |
| INPA-H 47580 | 0.37 | 0.34 | 0.49 | 0.37 | 0.61 | 0.48 | 0.84 | 0.60 | 0.80 | 0.55 | 4.58 | 3.91 | 3.87 | 1.74 | 2.81 | 3.19 | 0.40 | 0.34 | 0.70 | 0.50 | 0.99 | 0.68 | 0.89 | 0.64 |
| INPA-H 47581 | 0.33 | 0.28 | 0.50 | 0.46 | 0.63 | 0.46 | 0.83 | 0.68 | 0.72 | 0.55 | 4.35 | 3.74 | 3.98 | 1.56 | 2.86 | 3.05 | 0.48 | 0.38 | 0.61 | 0.45 | 0.87 | 0.67 | 0.78 | 0.59 |
| INPA-H 47582 | 0.34 | 0.33 | 0.45 | 0.38 | 0.64 | 0.48 | 0.85 | 0.67 | 0.74 | 0.65 | 4.70 | 3.99 | 4.21 | 1.70 | 2.88 | 3.19 | 0.48 | 0.40 | 0.60 | 0.47 | 0.85 | 0.63 | 0.74 | 0.57 |
| INPA-H 47583 | 0.37 | 0.35 | 0.47 | 0.43 | 0.57 | 0.47 | 0.75 | 0.60 | 0.67 | 0.56 | 5.23 | 4.19 | 4.85 | 1.99 | 3.09 | 3.62 | 0.41 | 0.34 | 0.56 | 0.39 | 0.86 | 0.57 | 0.77 | 0.57 |
| INPA-H 47584 | 0.41 | 0.36 | 0.41 | 0.40 | 0.68 | 0.48 | 0.84 | 0.71 | 0.82 | 0.63 | 4.53 | 3.86 | 4.45 | 1.72 | 2.96 | 3.43 | 0.51 | 0.44 | 0.71 | 0.60 | 1.00 | 0.78 | 0.92 | 0.71 |
| INPA-H 47586 | 0.38 | 0.37 | 0.47 | 0.42 | 0.61 | 0.48 | 0.84 | 0.63 | 0.76 | 0.59 | 4.79 | 3.97 | 4.20 | 1.67 | 2.90 | 3.38 | 0.40 | 0.36 | 0.62 | 0.43 | 0.89 | 0.63 | 0.81 | 0.60 |
| INPA-H 47587 | 0.32 | 0.30 | 0.38 | 0.34 | 0.53 | 0.44 | 0.62 | 0.55 | 0.60 | 0.51 | 4.42 | 3.94 | 4.05 | 1.64 | 2.87 | 3.07 | 0.36 | 0.36 | 0.52 | 0.47 | 0.73 | 0.63 | 0.65 | 0.55 |
| INPA-H 47588 | 0.34 | 0.37 | 0.47 | 0.39 | 0.63 | 0.52 | 0.87 | 0.65 | 0.79 | 0.61 | 4.09 | 3.68 | 3.97 | 1.57 | 2.69 | 2.99 | 0.40 | 0.37 | 0.65 | 0.51 | 0.99 | 0.77 | 0.83 | 0.58 |
| INPA-H 47589 | 0.31 | 0.33 | 0.45 | 0.38 | 0.66 | 0.50 | 0.85 | 0.63 | 0.73 | 0.58 | 4.23 | 3.92 | 4.12 | 1.62 | 2.83 | 3.18 | 0.44 | 0.40 | 0.63 | 0.47 | 0.94 | 0.65 | 0.81 | 0.60 |
| INPA-H 47590 | 0.38 | 0.34 | 0.51 | 0.38 | 0.68 | 0.50 | 0.83 | 0.62 | 0.76 | 0.59 | 4.74 | 4.07 | 4.36 | 1.78 | 2.93 | 3.33 | 0.31 | 0.29 | 0.67 | 0.47 | 0.91 | 0.74 | 0.83 | 0.66 |
| MPEG 45228 | 0.40 | 0.39 | 0.51 | 0.44 | 0.69 | 0.57 | 0.80 | 0.64 | 0.77 | 0.58 | 4.84 | 4.02 | 4.35 | 1.88 | 3.04 | 3.23 | 0.48 | 0.43 | 0.71 | 0.53 | 0.85 | 0.68 | 0.78 | 0.57 |
| MPEG 45229 | 0.35 | 0.31 | 0.44 | 0.36 | 0.58 | 0.51 | 0.80 | 0.61 | 0.76 | 0.55 | 4.62 | 3.71 | 3.91 | 1.71 | 2.65 | 3.06 | 0.32 | 0.26 | 0.71 | 0.49 | 0.98 | 0.70 | 0.94 | 0.68 |
| INPA-H 47591 | 0.28 | 0.27 | 0.40 | 0.35 | 0.60 | 0.51 | 0.76 | 0.61 | 0.55 | 0.48 | 4.11 | 3.45 | 3.70 | 1.51 | 2.60 | 2.73 | 0.34 | 0.29 | 0.64 | 0.50 | 0.81 | 0.56 | 0.77 | 0.61 |
