# Supplementary material for: A remarkable new blue Ranitomeya species (Anura: Dendrobatidae) with copper metallic legs from open forests of Juruá River Basin, Amazonia
Source: PLoS One. 2025 May 14;20(5):e0321748. doi: 10.1371/journal.pone.0321748 (PMC12077741; doi:10.1371/journal.pone.0321748)
Supplement: S2 Table — Abbreviations: vouchers: INPA-H, Instituto Nacional de Pesquisas da Amazônia; MPEG, Museu Paraense Emílio Goeldi; FNJV, Fonoteca Neotropical Jacques Vielliard; AT, air temperature (ºC); NN, number of notes per call; CD, call duration (ms); SBC, silence between calls (s); ND, note duration (ms); SBN, silence between notes (ms); LF, minimum frequency (Hz); HF, maximum frequency (Hz); and DF, dominant frequency (Hz). (DOCX) [file pone.0321748.s002.docx]

**S2 Table. Acoustic parameters of call of *Ranitomeya aetherea* sp. nov. Advertisement and courtship (bold) calls.**

Abbreviations: vouchers: INPAH, Instituto Nacional de Pesquisas da Amazônia; MPEG, Museu Paraense Emílio Goeldi; FNJV, Fonoteca Neotropical Jacques Vielliard; AT, air temperature (ºC); NN, number of notes per call; CD, call duration (ms); SBC, silence between calls (s); ND, note duration (ms); SBN, silence between notes (ms); LF, minimum frequency (Hz); HF, maximum frequency (Hz); and DF, dominant frequency (Hz).

| Voucher | | AT | NN | CD | SBC | ND | SBN | NR | LF | HF | DF |
| --- | --- | --- | --- | --- | --- | --- | --- | --- | --- | --- | --- |
| specimen | call recorded |  |  |  |  |  |  |  |  |  |  |
| **MPEG 45225** | FNJV 0124340 | 24.5 | 29 | 919 | 17.1 | 7.9 | 25.3 | 31.6 | 5,032 | 5,796 | 5,513 |
| **MPEG 45225** | FNJV 0124340 | 24.5 | 35 | 1,188 | 17.6 | 8 | 25 | 29.4 | 4,981 | 6,036 | 5,514 |
| **MPEG 45225** | FNJV 0124340 | 24.5 | 41 | 1,345 | 14.5 | 8.3 | 24.8 | 30.5 | 4,104 | 5,954 | 5,515 |
| **MPEG 45225** | FNJV 0124340 | 24.5 | 35 | 1,224 | 3.8 | 7.6 | 25.8 | 28.6 | 3,249 | 6,415 | 5,516 |
| **MPEG 45225** | FNJV 0124340 | 24.5 | 31 | 1,083 | 6.7 | 7.2 | 25.5 | 28.6 | 3,427 | 6,142 | 5,517 |
| **MPEG 45225** | FNJV 0124340 | 24.5 | 33 | 1,102 | 9.3 | 7.5 | 26.5 | 29.9 | 3,309 | 6,242 | 5,518 |
| **MPEG 45225** | FNJV 0124340 | 24.5 | 38 | 1,160 | 4.9 | 8 | 26.7 | 32.7 | 3,322 | 6,260 | 5,426 |
| **MPEG 45225** | FNJV 0124340 | 24.5 | 28 | 967 | 16 | 8.2 | 26 | 28.9 | 3,040 | 7,102 | 5,340 |
| **MPEG 45225** | FNJV 0124340 | 24.5 | 39 | 1,284 | 9.3 | 8.1 | 25.9 | 30.4 | 3,540 | 6,310 | 5,426 |
| **MPEG 45225** | FNJV 0124340 | 24.5 | 34 | 1,018 | - | 7.9 | 26.1 | 33.4 | 3,372 | 6,142 | 5,426 |
| INPA-H 47573 | FNJV 0124341 | 27.3 | 26 | 725 | 11.1 | 10.7 | 17.8 | 35.9 | 5,354 | 6,197 | 5,771 |
| INPA-H 47573 | FNJV 0124341 | 27.3 | 33 | 958 | 8.9 | 11.4 | 18.1 | 34.4 | 5,446 | 6,374 | 5,857 |
| INPA-H 47573 | FNJV 0124341 | 27.3 | 31 | 881 | 4.7 | 11.5 | 18.2 | 35.2 | 5,227 | 6,405 | 5,857 |
| INPA-H 47573 | FNJV 0124341 | 27.3 | 27 | 747 | 14.7 | 10.5 | 17.9 | 36.1 | 5,427 | 6,275 | 5,857 |
| INPA-H 47573 | FNJV 0124341 | 27.3 | 35 | 1,001 | 13.4 | 11.1 | 18.1 | 34.8 | 5,413 | 6,359 | 5,587 |
| INPA-H 47573 | FNJV 0124341 | 27.3 | 31 | 874 | - | 10.6 | 18.4 | 35.5 | 5,527 | 6,316 | 5,943 |
| INPA-H 47575 | FNJV 0124342 | 26.5 | 22 | 714 | - | 11 | 22.4 | 30.8 | 5,474 | 6,352 | 5,943 |
| INPA-H 47576 | FNJV 0124343 | 26.2 | 25 | 793 | 9.4 | 10.1 | 24.1 | 31.5 | 5,135 | 6,132 | 5,513 |
| INPA-H 47576 | FNJV 0124343 | 26.2 | 27 | 831 | - | 10.2 | 22.9 | 32.5 | 5,179 | 6,132 | 5,513 |
| INPA-H 47578 | FNJV 0124344 | 26 | 29 | 810 | 9 | 11 | 17.9 | 35.8 | 5,467 | 6,304 | 5,857 |
| INPA-H 47578 | FNJV 0124344 | 26 | 26 | 703 | 33.6 | 10.7 | 17.6 | 37 | 5,406 | 6,347 | 5,857 |
| INPA-H 47578 | FNJV 0124344 | 26 | 26 | 717 | 15.7 | 10.4 | 17.8 | 36.3 | 5,476 | 6,225 | 5,857 |
| INPA-H 47578 | FNJV 0124344 | 26 | 28 | 768 | 26.6 | 10.8 | 17.7 | 36.5 | 5,367 | 6,305 | 5,857 |
| INPA-H 47578 | FNJV 0124344 | 26 | 29 | 808 | - | 10.4 | 18.1 | 35.9 | 5,407 | 6,351 | 5.857 |
| INPA-H 47581 | FNJV 0124345 | 26.4 | 21 | 628 | 14.8 | 11.1 | 20.4 | 33.4 | 5,197 | 6,284 | 5,771 |
| INPA-H 47581 | FNJV 0124345 | 26.4 | 21 | 640 | 20.7 | 11.5 | 20 | 32.8 | 5,106 | 6,177 | 5,771 |
| INPA-H 47581 | FNJV 0124345 | 26.4 | 20 | 619 | 28.8 | 11.7 | 20.2 | 32.3 | 5,301 | 6,160 | 5,771 |
| INPA-H 47581 | FNJV 0124345 | 26.4 | 16 | 490 | - | 11.4 | 18.1 | 32.7 | 4,907 | 6,074 | 5,771 |
| INPA-H 47586 | FNJV 0124346 | 25.8 | 18 | 540 | - | 15.6 | 15.9 | 33.3 | 5,550 | 6,371 | 6,029 |
| INPA-H 47587 | FNJV 0124347 | 25.6 | 23 | 670 | 25.6 | 12.4 | 17.4 | 34.3 | 4,743 | 6,432 | 5,857 |
| INPA-H 47587 | FNJV 0124347 | 25.6 | 24 | 722 | 7.6 | 12.2 | 18.6 | 33.3 | 4,941 | 6,376 | 5,857 |
| INPA-H 47587 | FNJV 0124347 | 25.6 | 24 | 675 | 11.4 | 11.1 | 17.8 | 35.6 | 5,141 | 6,289 | 5,857 |
| INPA-H 47587 | FNJV 0124347 | 25.6 | 24 | 699 | - | 12.1 | 17.7 | 34.3 | 5,054 | 6,376 | 5,857 |
