# Supplementary figures and images for: A remarkable new blue Ranitomeya species (Anura: Dendrobatidae) with copper metallic legs from open forests of Juruá River Basin, Amazonia
Source: PLoS One. 2025 May 14;20(5):e0321748. doi: 10.1371/journal.pone.0321748 (PMC12077741; doi:10.1371/journal.pone.0321748)

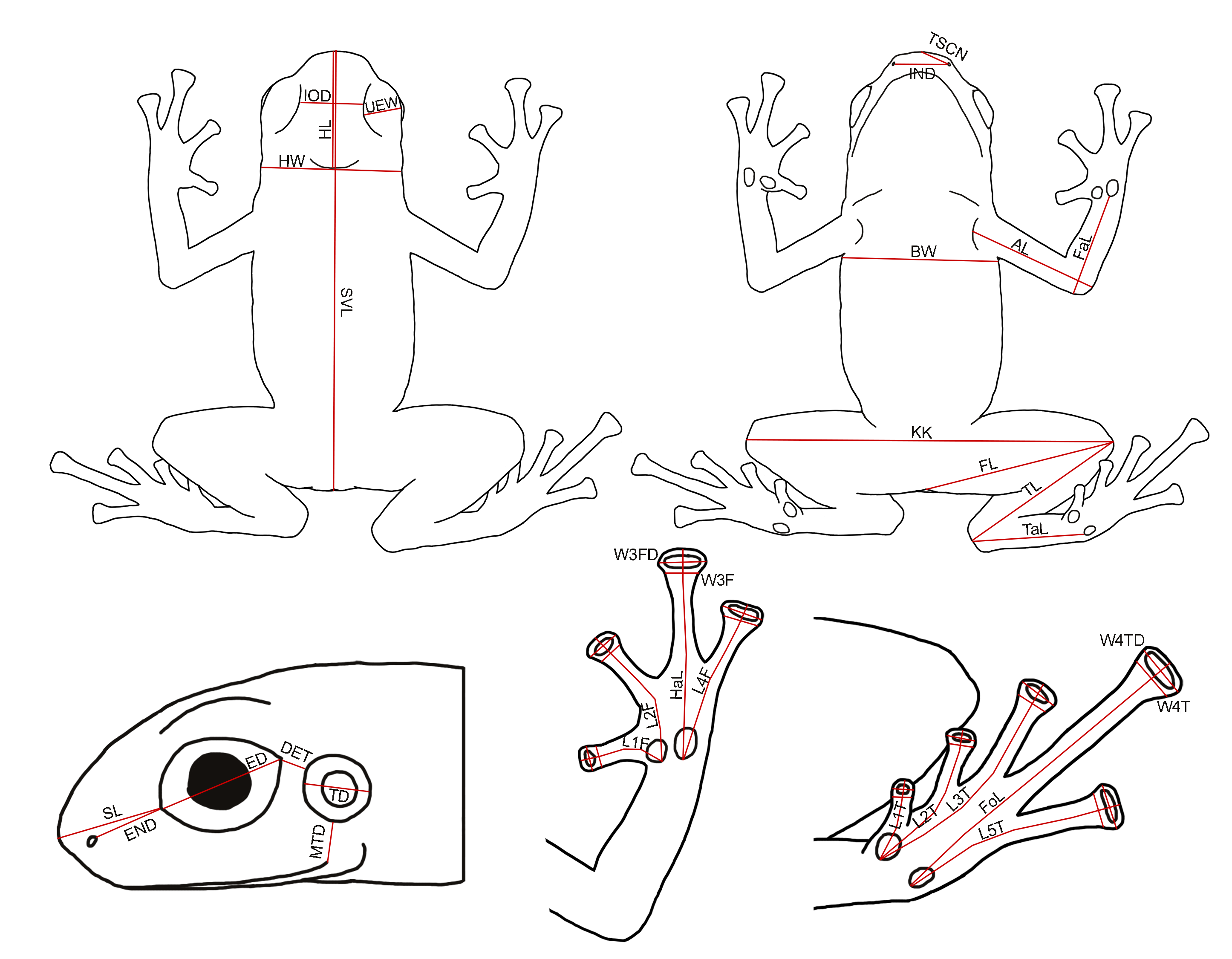

Supplement: S1 Fig — (TIF) [file pone.0321748.s007.tif]

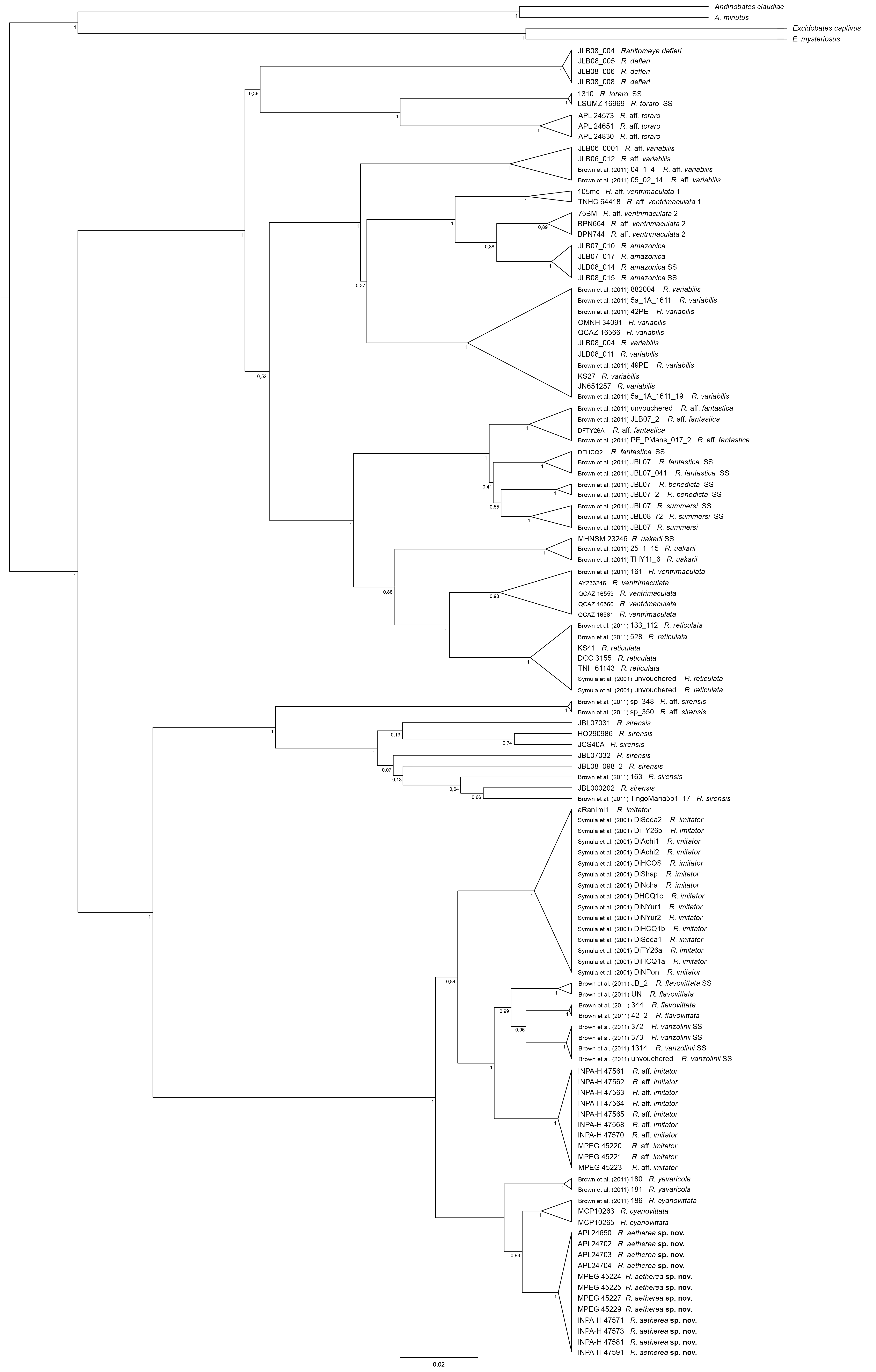

Supplement: S2 Fig — Bayesian inference tree inferred with 16S, 12S, COI and CytB. Non-parametric bootstrap support is shown close to nodes. (TIF) [file pone.0321748.s008.tif]
